# Improved Conditions for the Visible-Light Driven Hydrocarboxylation by Rh(I) and Photoredox Dual Catalysts Based on the Mechanistic Analyses

**DOI:** 10.3389/fchem.2019.00371

**Published:** 2019-05-22

**Authors:** Kei Murata, Nobutsugu Numasawa, Katsuya Shimomaki, Jun Takaya, Nobuharu Iwasawa

**Affiliations:** Department of Chemistry, Tokyo Institute of Technology, Tokyo, Japan

**Keywords:** carboxylation, CO_2_ fixation, photoredox catalyst, rhodium catalyst, visible light

## Abstract

The improved catalytic conditions and detailed reaction mechanism of the visible-light driven hydrocarboxylation of alkenes with CO_2_ by the Rh(I) and photoredox dual catalysts were investigated. The use of the benzimidazoline derivative, BI(OH)H, as a sacrificial electron donor was found to increase the yield of the hydrocarboxylated product by accelerating the reduction process. In addition, the incorporation of the cyclometalated Ir(III) complex as a second photosensitizer with [Ru(bpy)_3_]^2+^ photosensitizer also resulted in the promotion of the reduction process, supporting that the catalytic cycle includes two photochemical elementary processes: photoinduced electron and energy transfers.

## Introduction

Catalytic hydrocarboxylation of unsaturated hydrocarbons with CO_2_ is one of the promising methods for the CO_2_ fixation (Luan and Ye, 2018; Yan et al., 2018; for recent reviews, see: Tortajada et al., 2018). The most common strategy to accomplish the hydrocarboxylation is to utilize a metal hydride complex as an active species. However, in these reactions, more than a stoichiometric amount of highly active, metallic reductants such as ZnEt_2_, AlEt_3_, or hydrosilanes are usually required to promote the reduction process in the catalytic cycle (Takaya and Iwasawa, 2008; Williams et al., 2008; Fujihara et al., 2011; Li et al., 2011; Hayashi et al., 2015; Wang et al., 2015; Zhu et al., 2015; Kawashima et al., 2016). In order to realize a more efficient and environmentally-friendly system, the reaction which necessitates just a catalytic amount of metallic reagents is highly desirable. Meanwhile, the photochemical reduction process has been widely employed in the field of artificial photosynthesis, such as photocatalytic hydrogen generations (for review, see Esswein and Nocera, 2007) and CO_2_ reductions (for reviews, see Morris et al., 2009; Doherty et al., 2010; Takeda and Ishitani, 2010) in homogeneous systems. In these reactions, transition-metal catalysts are combined with redox photosensitizers and sacrificial electron donors to drive the multielectron transfer processes under visible-light irradiation. When the electron transfer is accompanied by the proton transfer, metal hydrides can act as an active species in the catalytic cycle (for reviews, see Stoll et al., 2015; Adams et al., 2018). Although transition-metal/photoredox dual catalysis has been actively studied in the field of organic synthesis (for recent reviews, see Fabry and Rueping, 2016; Skubi et al., 2016; Twilton et al., 2017), few examples have been reported for catalytic organic transformations driven by photochemically-generated metal hydrides (Ghosh et al., 2015).

**Figure 1:**
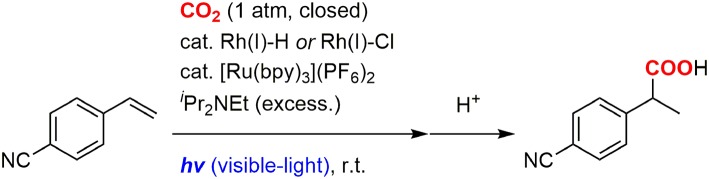
The photocatalytic hydrocarboxylation of 4-cyanostyrene.

We recently developed the visible-light driven hydrocarboxylation of alkenes with CO_2_ for the first time by means of the photochemical generation of Rh(I) hydride species (Murata et al., 2017). 4-Cyanostyrene was transformed to the branched hydrocarboxylated product by using a Rh(I) hydride or chloride complex as a carboxylation catalyst, [Ru(bpy)_3_]^2+^ as a photoredox catalyst, ^*i*^Pr_2_NEt as a sacrificial electron donor, with visible-light irradiation under CO_2_ atmosphere at room temperature (Figure 1). The photoredox catalysis made it possible to take electrons from tertiary amines and drive the reduction process without using a metallic reductant. Since then, several photoredox-catalyzed hydrocarboxylation reactions of unsaturated hydrocarbons with CO_2_ have been reported by other groups (Seo et al., 2017b; Hou et al., 2018b; Meng et al., 2018). Concomitantly, difunctionalizations of alkenes such as thiocarboxylation (Ye et al., 2017), carbocarboxylation and silylcarboxylation (Yatham et al., 2017; Hou et al., 2018a) have also been developed by incorporating an appropriate radical precursor with CO_2_. Furthermore, in addition to unsaturated hydrocarbons, various substrates such as aryl and alkyl halides (Meng et al., 2017; Shimomaki et al., 2017), amines (Seo et al., 2017a), imines and enamides (Fan et al., 2018; Ju et al., 2018) have been carboxylated with CO_2_ by photoredox catalysis so far (for review, see Yeung, 2019). These examples demonstrated wide applicability of the photoinduced electron transfer to carboxylation reactions.

**Figure 2:**
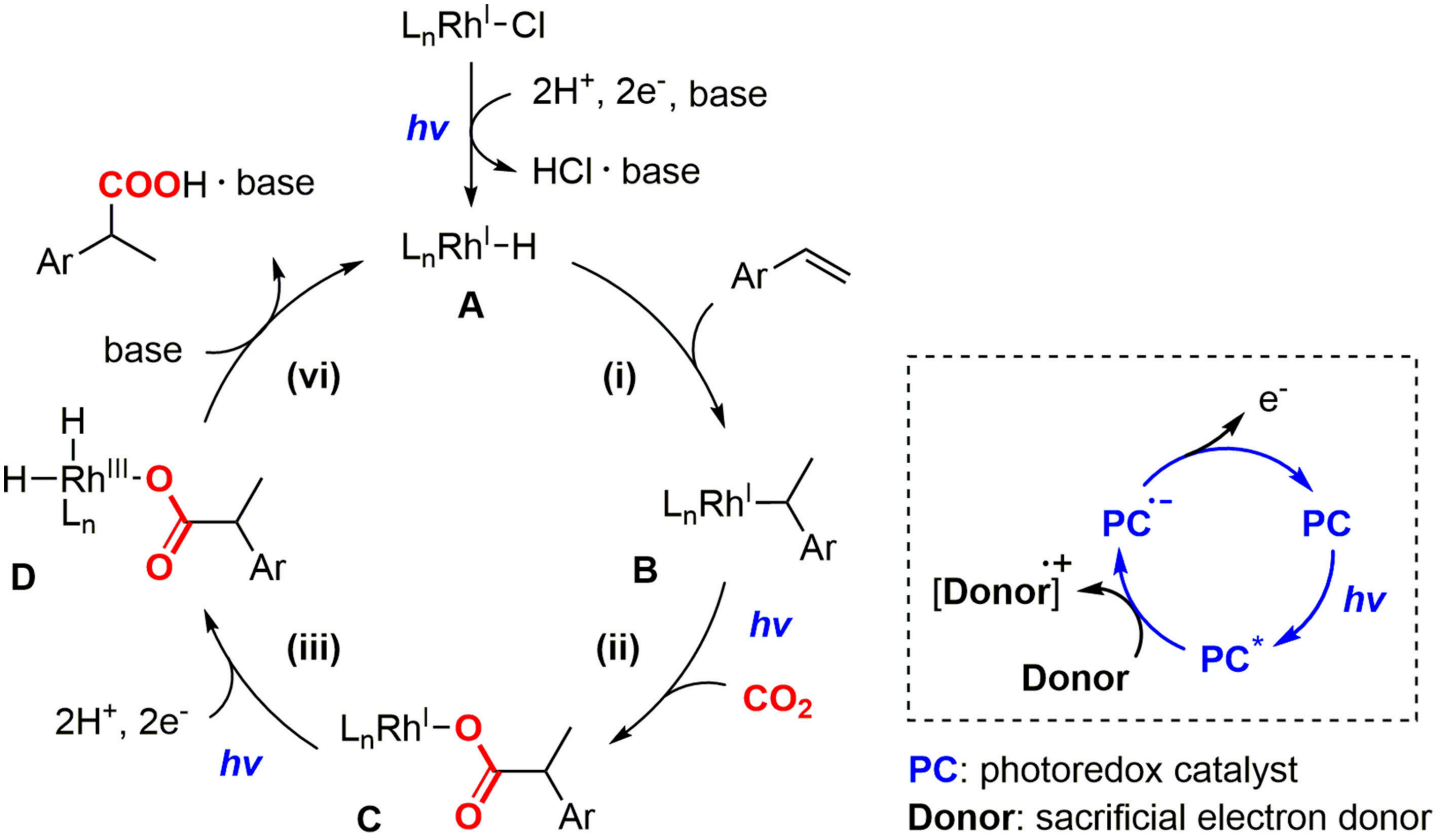
The proposed reaction mechanism of the photocatalytic hydrocarboxylation with the schematic representation of the reductive quenching cycle of a photoredox catalyst.

On the basis of our previous experiments, the reaction mechanism of the hydrocarboxylation by Rh(I) and photoredox dual catalysts was proposed as shown in Figure 2. Initially, the hydrometallation of a styrene derivative by Rh(I) hydride species **A** gave the Rh(I) benzyl species **B** (i), and the visible-light promoted nucleophilic addition to CO_2_ afforded the Rh(I) carboxylate species **C** (ii). Then, the reductive quenching cycle of [Ru(bpy)_3_]^2+^ with ^*i*^Pr_2_NEt mediated 2-electron, 2-proton transfers afforded the Rh(III) dihydride carboxylate species **D** (iii), followed by the base-promoted liberation of the carboxylated product to regenerate the active species **A** (iv). Although this reaction demonstrated fundamental aspects of the application of photochemical reduction processes to catalytic carboxylation reactions, there still has been room for improvement from the viewpoint of the applicability in organic synthesis: (i) The efficiency of the reaction was not very high. Good yield was obtained with 4-cyanostyrene and moderate yields were obtained with several other substrates. (ii) A large excess amount of a tertiary amine and long reaction time (>24 h) were necessary for completion of the reaction even for the reactive substrates, (iii) A significant amount of the hydrogenated product was produced as a byproduct. In order to resolve these problems, further screenings of the catalytic conditions were desired.

Herein, we explored the improved conditions of the visible-light driven hydrocarboxylation, and the catalytic efficiency was analyzed based on the detailed mechanistic study with a series of stoichiometric reactions of the rhodium intermediates. Through the investigation, the hydrocarboxylation was successfully improved by the alteration of the sacrificial electron donor or the incorporation of the second photosensitizer. The mechanistic study suggested that the promotion of the photochemical reduction process was crucial for the enhancement of the catalytic reaction.

## Results and Discussion

### Screening of Reaction Conditions

On the basis of our previous experiments in terms of the screening of catalytic conditions and the observation of the reaction intermediates under the catalytic conditions, the followings were demonstrated: (i) As a carboxylation catalyst, Rh(I) hydride or chloro complexes with triarylphosphines were applicable. In particular, the μ-chloro bridged Rh(I) dimer [Rh(P(4-CF_3_C_6_H_4_)_3_)_2_Cl]_2_ (**4**) was the most effective catalyst. (ii) When **4** was employed under the catalytic conditions, the resting state was the corresponding Rh(I) carboxylate complex, indicating that the rate-determining step was its transformation to the Rh(I) hydride species. This result suggested that the promotion of the reduction process was crucial for the improvement of the catalytic reaction. According to these considerations, the reaction conditions were screened in terms of the photoredox catalyst and sacrificial electron donor, which would have taken an important part in the reductive quenching cycle.

#### Photoredox Catalyst

Photoredox catalysts were initially screened by performing the reaction of 4-cyanostyrene (**1a**) in a mixture of 2.0 mol% of a photosensitizer, 3.5 mol% of **4** and 4.0 equiv. of ^*i*^Pr_2_NEt under a CO_2_ atmosphere at room temperature (Table 1). In the case of [Ru(bpy)_3_](PF_6_)_2_ ($E_{1/2}^{\text{I}{\text{I}}^{*}/I}$ = +0.77 V, $E_{1/2}^{\text{II}/\text{I}}$ = −1.33 V vs. SCE) (Kalyanasundaram, 1982) as a photoredox catalyst, the hydrocarboxylated (**2a**) and hydrogenated (**3a**) products were obtained in 54 and 25% yields, respectively, after visible-light irradiation for 24 h (Table 1, entry 1). A small amount of polymerized product of **1a** was also produced as byproduct. Though no other photosensitizers overcame this activity, the yield of **2a** was found to be strongly dependent on the photoredox catalyst. For instance, when [Ru(bpz)_3_](PF_6_)_2_ ($E_{1/2}^{\text{I}{\text{I}}^{*}/I}$ = +1.45 V, $E_{1/2}^{\text{II}/\text{I}}$ = −0.80 V vs. SCE) (Crutchley and Lever, 1980) or *fac*-Ir(ppy)_3_ ($E_{1/2}^{\text{II}{\text{I}}^{*}/II}$ = +0.31 V, $E_{1/2}^{\text{III}/\text{II}}$ = −2.19 V vs. SCE) (Flamigni et al., 2007) was employed, the yields substantially decreased compared to [Ru(bpy)_3_](PF_6_)_2_ (Table 1, entries 3, 6). On the other hand, when using [Ir(dF(CF_3_)ppy)_2_(dtbbpy)](PF_6_) ($E_{1/2}^{\text{II}{\text{I}}^{*}/II}$ = +1.21 V, $E_{1/2}^{\text{III}/\text{II}}$ = −1.37 V vs. SCE) or [Ir(ppy)_2_(dtbbpy)](PF_6_) ($E_{1/2}^{\text{II}{\text{I}}^{*}/II}$ = +0.66 V, $E_{1/2}^{\text{III}/\text{II}}$ = −1.51 V vs. SCE) (Lowry et al., 2005), moderate yields were obtained (Table 1, entry 4, 5). These results indicated that both sufficient oxidizing ability of the excited state and reducing ability of the one-electron reduced species are at least necessary for the photosensitizer. However, the detailed dependency was not simple, as other factors such as absorption properties, excited-state energies and photochemical stability of the photosensitizer could also affect the catalytic performances. Meanwhile, the screenings of additives with [Ru(bpy)_3_]^2+^ photosensitizer demonstrated that the addition of Cs_2_CO_3_ as an inorganic base significantly improved the yield of the hydrocarboxylated product by suppressing the formation of the hydrogenated byproduct: the yield of **2a** increased to 67% while the yield of **3a** decreased to 1% (Table 1, entry 11).

**Table 1:** Optimization of the reaction conditions of the hydrocarboxylation of 4-cyanostyrene.

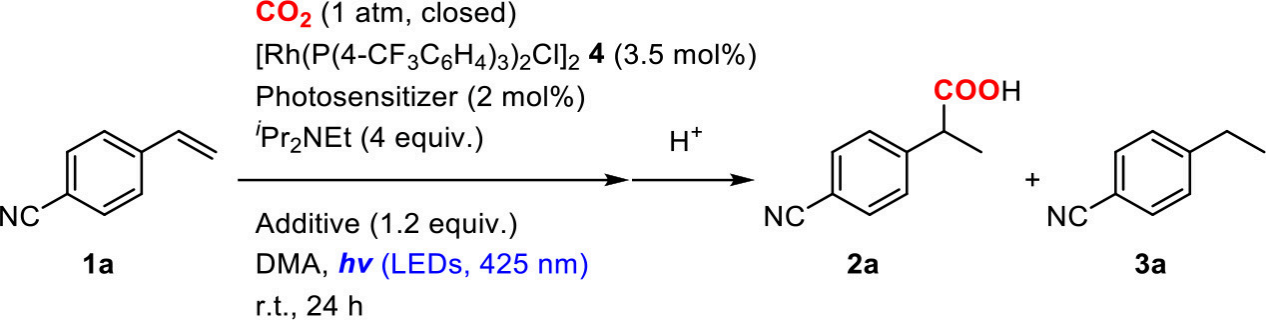					
**Entry**	**Photosensitizer**	**Additive**	**Conv. /%**	**Yield/%**	
				**2a^a^**	**3a^b^**
1	[Ru(bpy)_3_](PF_6_)_2_	–	95	54	25
2	[Ru(dmbpy)_3_](PF_6_)_2_	–	92	37	14
3	[Ru(bpz)_3_](PF_6_)_2_	–	24	n.d.	n.d.
4	[Ir(dF(CF_3_)ppy)_2_ (dtbpy)](PF_6_)	–	>99	44	8
5	[Ir(ppy)_2_(dtbbpy)](PF_6_)	–	>99	27	trace
6	*fac*-Ir(ppy)_3_	–	98	17	2
7^c^	[Ru(bpy)_3_](PF_6_)_2_	–	72	33	8
8^d^	[Ru(bpy)_3_](PF_6_)_2_	–	95	46	23
9	[Ru(bpy)_3_](PF_6_)_2_	Na_2_CO_3_	89	43	trace
10	[Ru(bpy)_3_](PF_6_)_2_	K_2_CO_3_	87	46	trace
11	[Ru(bpy)_3_](PF_6_)_2_	Cs_2_CO_3_	70	67	1

a *NMR yield*.

b *GC yield*.

c *1 mol% of [Ru(bpy)3](PF_6_)_2_*.

d *5 mol% of [Ru(bpy)3](PF_6_)_2_*.

#### Sacrificial Electron Donor and Additives

Sacrificial electron donors were then screened in the presence of an excess amount of Cs_2_CO_3_. In order to highlight the reactivity, a less reactive alkene, 3,5-bis(trifluoromethyl)styrene (**1b**), was used as a substrate. The reactions of **1b** were performed in a mixture of 2.0 mol% of [Ru(bpy)_3_](PF_6_)_2_, 3.5 mol% of **4**, 4.0 equiv. of sacrificial electron donor and 1.2 equiv. of Cs_2_CO_3_ under a CO_2_ atmosphere at room temperature (Table 2). When ^*i*^Pr_2_NEt was employed as a sacrificial electron donor, 32% yield of the hydrocarboxylated product (**2b**) and a trace amount of the hydrogenated product (**3b**) were obtained after visible-light irradiation for 12 h (Table 2, entry 1). Although the use of TEOA (triethanolamine) slightly increased the yield of **2b**, the formation of **3b** became pronounced probably due to the increase of proton concentration (Table 2, entry 3). On the other hand, the use of BI(OH)H (1,3-dimethyl-2-(*o*-hydroxyphenyl)-2,3-dihydro-1*H*-benzo[*d*]imidazole) successfully accelerated the hydrocarboxylation and increased the yield of **2b** considerably with maintaining the low yield of **3b** (Table 2, entry 5). Furthermore, the incorporation of BI(OH)H made it possible to reduce the amounts of the photoredox catalyst and the sacrificial electron donor: the use of only 1.0 mol% of [Ru(bpy)_3_](PF_6_)_2_ and 2.0 equiv. of BI(OH)H gave 70% yield of **2b** (Table 2, entry 7). When the reaction was performed under an Ar atmosphere in the presence of Cs_2_CO_3_, no hydrocarboxylated product **2b** was obtained. This result confirmed that the carbonate did not work as a source of CO_2_ in the present reaction (Table 2, entry 9). BI(OH)H has been known to work as a 2-electron, 2-proton donor with high reducing ability in redox photosensitizing reactions (Hasegawa et al., 2005, 2006; Tamaki et al., 2015). Since a tertiary amine contributed to the reductive quenching cycle of [Ru(bpy)_3_]^2+^, the increase in the yield of **2b** was attributed to the promotion of the reduction process of the Rh(I) carboxylate species, which was the rate-determining step in the hydrocarboxylation. These results indicated that the redox property of a sacrificial electron donor is one of the crucial factors for the efficient promotion of the reaction.

**Table 2:** Optimization of the reaction conditions of the hydrocarboxylation of 3,5-bis(trifluoromethyl)styrene.

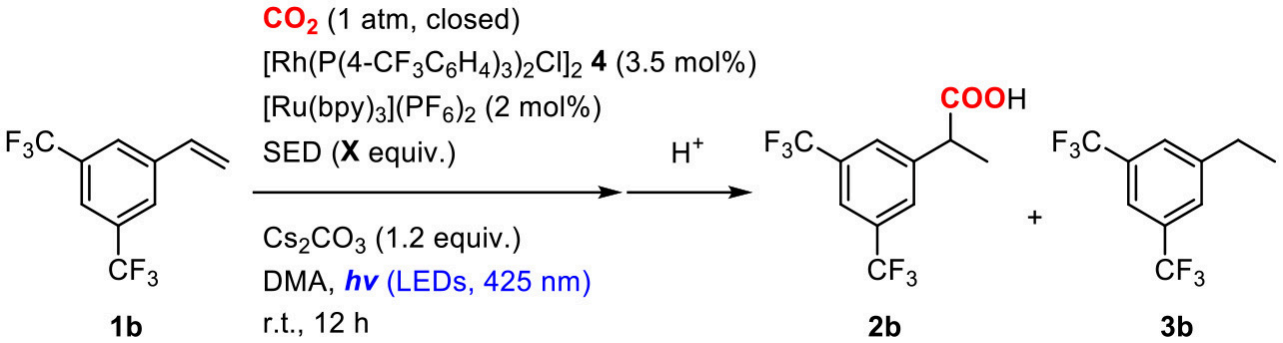					
**Entry**	**SED**	**X**	**Conv. /%**	**Yield/%**	
				**2b^a^**	**3b^b^**
1	*^*i*^*Pr_2_NEt	4.0	52	32	Trace
2	Et_3_N	4.0	16	4	n.d.
3	TEOA	4.0	>99	42	26
4	BIH	4.0	47	27	5
5	BI(OH)H	4.0	>99	63	5
6	BI(OH)H	2.0	>99	67	9
7^c^	BI(OH)H	2.0	>99	70^e^	3
8^c^	BI(OH)H	1.2	79	63	Trace
9^d^	BI(OH)H	2.0	>99	n.d.	34
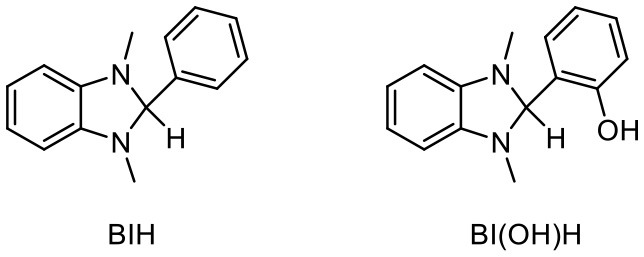					

a *NMR yield*.

b *GC yield*.

c *1 mol% of [Ru(bpy)_3_](PF_6_)_2_ was used*.

d *Without CO_2_, under an Ar atmosphere*.

e *Isolated yield was 63%*.

#### Generality of the Hydrocarboxylation Under the Improved Conditions

Based on the improved conditions using **1b** as discussed above, the generality of the hydrocarboxylation was examined using various alkene substrates. 1.0 mol% of [Ru(bpy)_3_](PF_6_)_2_, 3.5 mol% of **4**, 2.0 equiv. of BI(OH)H and 1.2 equiv. of Cs_2_CO_3_ were employed for the hydrocarboxylation (Table 3). In the cases of using styrenes with an electron-withdrawing group such as **1a** and 4-methoxycarbonyl styrene (**1d**), the reaction was almost completed after irradiation for 12 h, and the yields of the corresponding hydrocarboxylated products were significantly improved compared with those obtained in the previous conditions where 2.0 mol% of [Ru(bpy)_3_](PF_6_)_2_ and 4.0 equiv. of ^*i*^Pr_2_NEt were employed. Moreover, 4-trifluoromethyl styrene (**1c**) and non-substituted styrene (**1e**), which exhibited quite low reactivities in the previous conditions, did react to afford significant amounts of the corresponding hydrocarboxylated products though the yields were still not sufficiently high. The yields of the hydrocarboxylated products were also improved in the case of alkyl acrylates (**1f** and **1g**). Consequently, the introduction of BI(OH)H electron donor with Cs_2_CO_3_ base successfully resulted in the increase in the yields of the present hydrocarboxylation reaction.

**Table 3:**
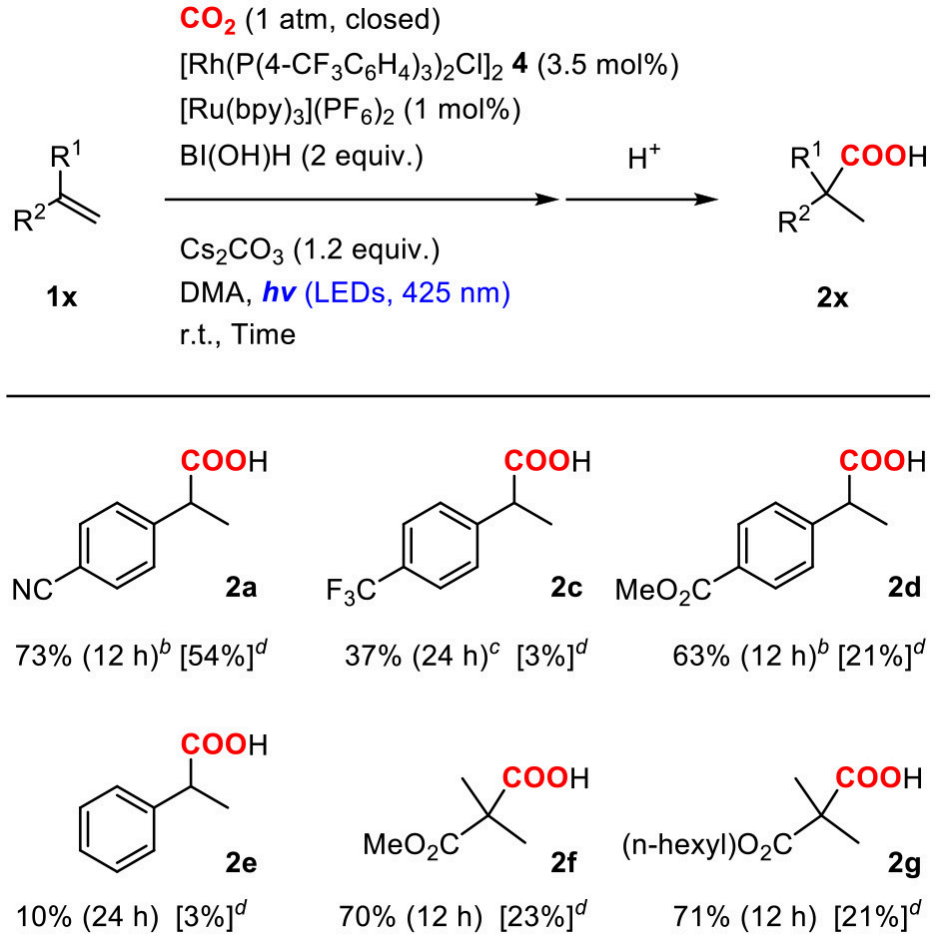
Generality of the hydrocarboxylation of alkenes^a^.

a *NMR yield*.

*^b^Isolated yield after methyl esterification: 57% (**2a**), 59% (**2d**)*.

^c^With 2 mol% of [Ru(bpy)_3_](PF_6_)_2._

*^d^Yield obtained by the previous conditions with [Ru(bpy)_3_](PF_6_)_2_ (2 mol%), **4** (3.5 mol%)*.

*^i^Pr_2_NEt (4 equiv.) and irradiation for 24 h*.

### Mechanistic Study

In order to reveal the reaction mechanism of the photocatalytic hydrocarboxylation, the stoichiometric reactions of the possible rhodium intermediates, which corresponded to each elementary step in the proposed catalytic cycle, were examined.

#### Rh Hydride Formation

**Figure 3:**
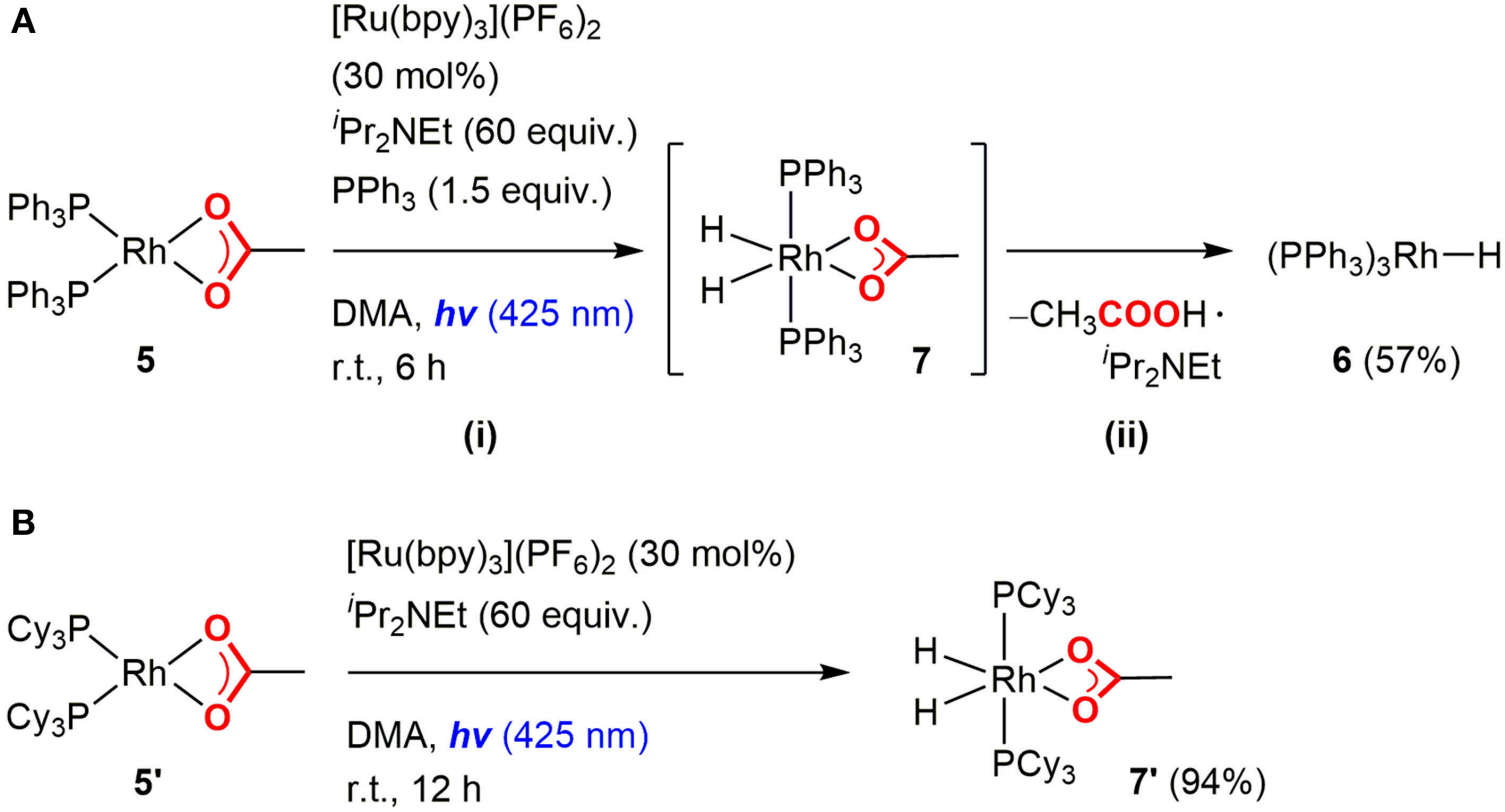
Rh hydride formation from the Rh(I) acetate complexes with **(A)** triphenylphosphines and **(B)** tricyclohexylphosphines.

**Figure 4:**
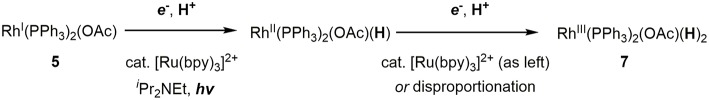
Proposed mechanism for the formation of the Rh(III) dihydride complex.

Initially, the Rh(I) hydride formation step was investigated using Rh(PPh_3_)_2_(OAc) (**5**) as a model complex of the Rh(I) carboxylate intermediate. The DMA solution of **5** was irradiated by visible-light in the presence of a catalytic amount of [Ru(bpy)_3_](PF_6_)_2_, an excess amount of ^*i*^Pr_2_NEt and 1.5 equivalent of PPh_3_. After visible-light irradiation for 6 h, the Rh(I) monohydride complex, Rh(PPh)_3_H (**6**) was successfully obtained in 57% yield with the recovery of ca. 30% of **5** (based on ^1^H NMR using an internal standard) (Figure 3A; Supplementary Figure 1). Control experiments demonstrated that [Ru(bpy)_3_](PF_6_)_2_, ^*i*^Pr_2_NEt, and visible-light were all essential for the transformation. Since hydrogen evolution was not evident during the reaction, the contribution of gaseous hydrogen was excluded. Thus, this transformation was considered to proceed via (i) stepwise 2-electron, 2-proton transfers from the tertiary amine by the photoredox catalysis to give the Rh(III) dihydride carboxylate (**7**), and (ii) the base-assisted elimination of the carboxylic acid to give **6**. In terms of step (i), the similar mechanisms have been proposed in photocatalytic hydrogen generation systems by a Rh(I) catalyst (Stoll et al., 2015). The initial single electron transfer to the protonated form of **5** would give the Rh(II) carboxylate monohydride, and the following electron and proton transfers or disproportionation of the two Rh(II) hydride species would give **7** (Figure 4). The presence of the Rh(III) dihydride intermediate was also supported by the fact that Rh(PCy_3_)_2_(OAc) (**5**′) was transformed to Rh(PCy_3_)_2_(OAc)(H)_2_ (**7**′) almost quantitatively under the similar conditions although the reaction was relatively slow (Figure 3B; Supplementary Figure 2). In this case, PCy_3_ ligands with strong σ-donation were considered to stabilize the Rh(III) dihydride intermediate to inhibit the following elimination reaction.

**Figure 5:**
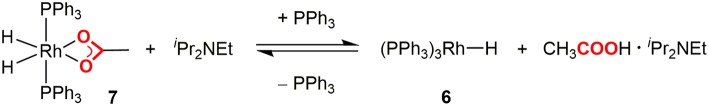
Equilibrium between the Rh(III) dihydride and Rh(I) monohydride complexes.

In order to confirm the carboxylic acid elimination step (ii), the reactivity of **7** was investigated in the presence of base. **7** was alternatively synthesized by the hydrogenation of **5** with H_2_, and was treated with an excess amount of ^*i*^Pr_2_NEt in the presence of PPh_3_ in C_6_D_6_. The reaction readily gave a mixture of **7** and **6** with liberation of [^*i*^Pr_2_NHEt]^+^[CH_3_COO]^−^. Furthermore, addition of a small excess amount of [^*i*^Pr_2_NHEt]^+^[CH_3_COO]^−^ to the C_6_D_6_ solution of **6** resulted in the quantitative formation of **7**. These results demonstrated that **7** was in equilibrium with **6** in the presence of ^*i*^Pr_2_NEt and PPh_3_ (Figure 5). When the treatment of **7** with ^*i*^Pr_2_NEt was similarly conducted in DMA, **6** was detected as a sole rhodium species in the reaction mixture, indicating that the equilibrium was almost completely shifted to the product side owing to the solvent effect of DMA.

The possible mechanisms for generation of the hydrogenated product were (i) 2-electron, 2-proton transfers to the Rh(I) benzyl intermediate by the photoredox catalysis to give Rh(III) benzyl dihydride intermediate, which would undergo reductive elimination of the hydrogenated product, and (ii) the alkene insertion to the Rh(III) dihydride intermediate and the successive reductive elimination. Both pathways could be inhibited by lowering proton concentrations, as proton transfers would become inefficient in the former, and the competing carboxylic acid elimination from the dihydride complex would be promoted in the latter. Therefore, the inhibition of the hydrogenated product formation by the addition of Cs_2_CO_3_ was attributed to the decrease of the proton concentration in the catalytic system.

The photochemical formation of Rh(I) monohydride species was also feasible by using Rh(I) chloride complex as a Rh(I) source. It was demonstrated by the fact that Wilkinson's type complex Rh(PPh_3_)_3_Cl was converted to Rh(PPh)_3_H (**6**) by visible-light irradiation in the presence of a catalytic amount of [Ru(bpy)_3_](PF_6_)_2_ and an excess amount of ^*i*^Pr_2_NEt. Therefore, the Rh(I) chloride complex was confirmed to work as a precursor of the Rh(I) hydride active species.

#### Hydrometalation and Carboxylation

**Figure 6:**
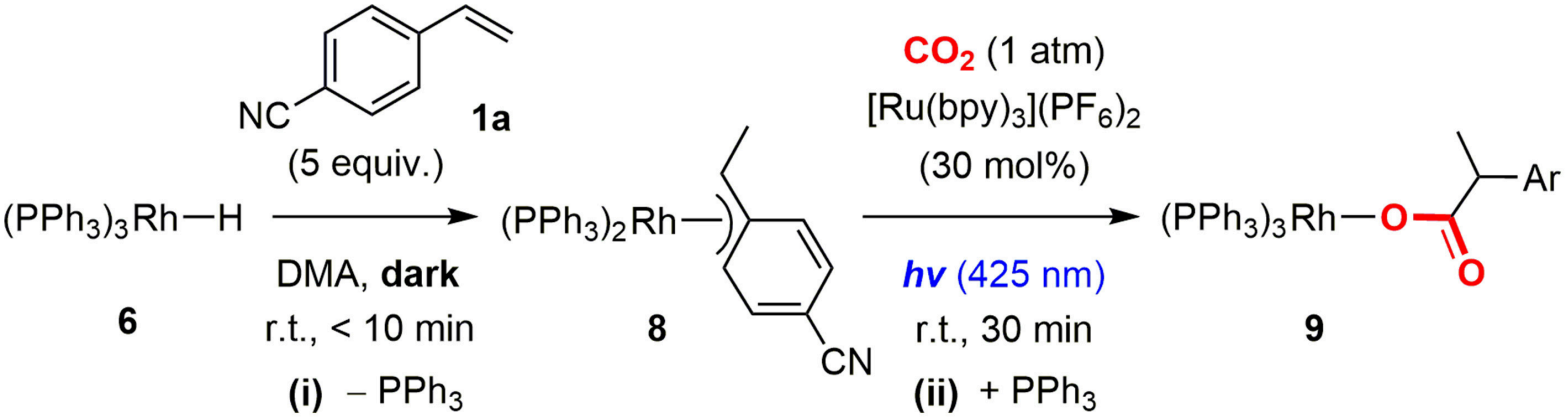
Hydrometallation of Rh(I) hydride complex and successive carboxylation of the Rh(I) π-benzyl complex with CO_2_.

Since the Rh(I) monohydride species was successfully generated from the Rh(I) carboxylate species by photoredox catalysis, the hydrometallation and subsequent carboxylation processes were then investigated to complete the catalytic cycle. Treatment of Rh(PPh)_3_H (**6**) with an excess amount of **1a** at room temperature readily formed the Rh(I) benzyl species, Rh(PPh_3_)_2_(η^3^-CHCH_3_(4-CNC_6_H_4_)) (**8**), almost quantitatively with the liberation of a PPh_3_ ligand (Figure 6i). The benzyl ligand in **8** was found to possess η^3^-coordination to the Rh(I) center based on NMR spectroscopic data (Werner et al., 1994). However, the attempt for isolation of **8** was not successful due to the presence of an equilibrium with **6**. Therefore, *in situ* generated **8** was directly used for the carboxylation step.

**Figure 7:**
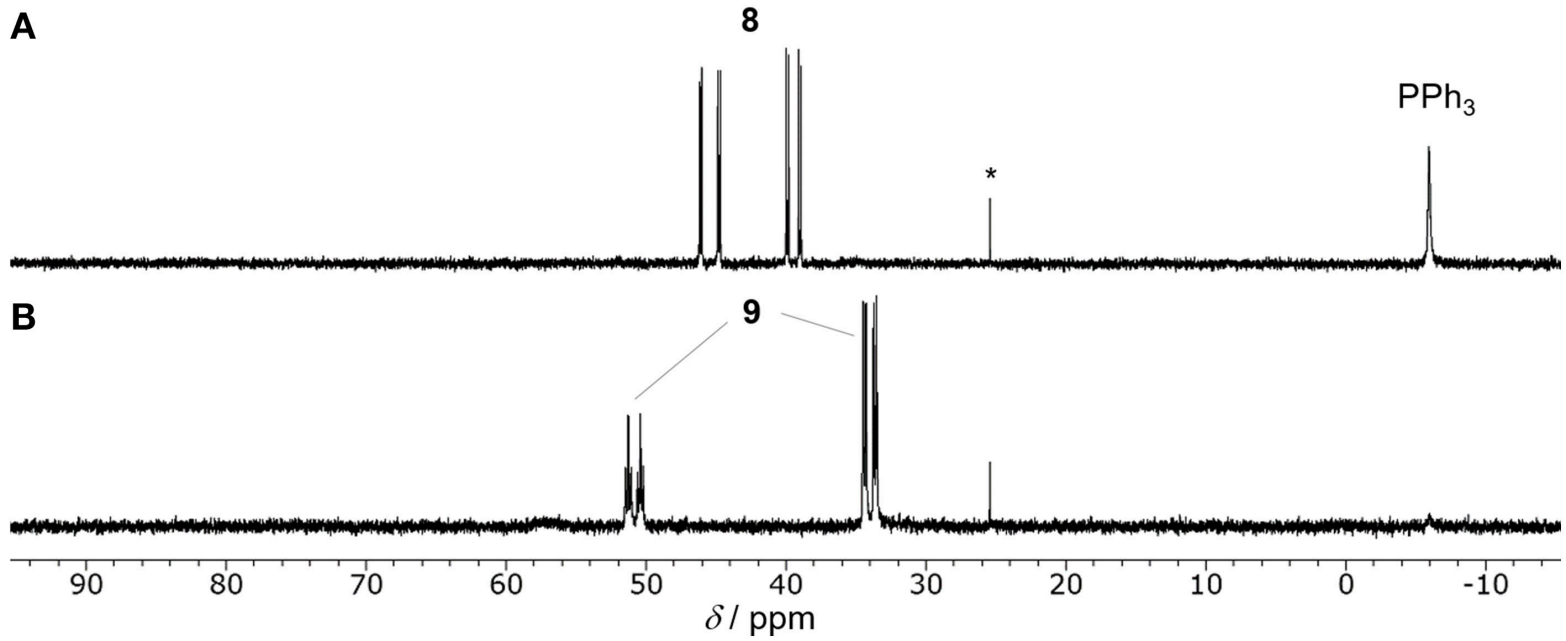
^31^P{^1^H} NMR spectra (202 MHz, DMA, −15°C) of the reactions of **8** under CO_2_ atmosphere in the presence of [Ru(bpy)_3_](PF_6_)_2_ (30 mol%) **(A)** after kept in dark, **(B)** after visible-light irradiation (λ_irr._ = 425 nm) for 30 min. ^*^(O=)PPh_3_.

**Figure 8:**
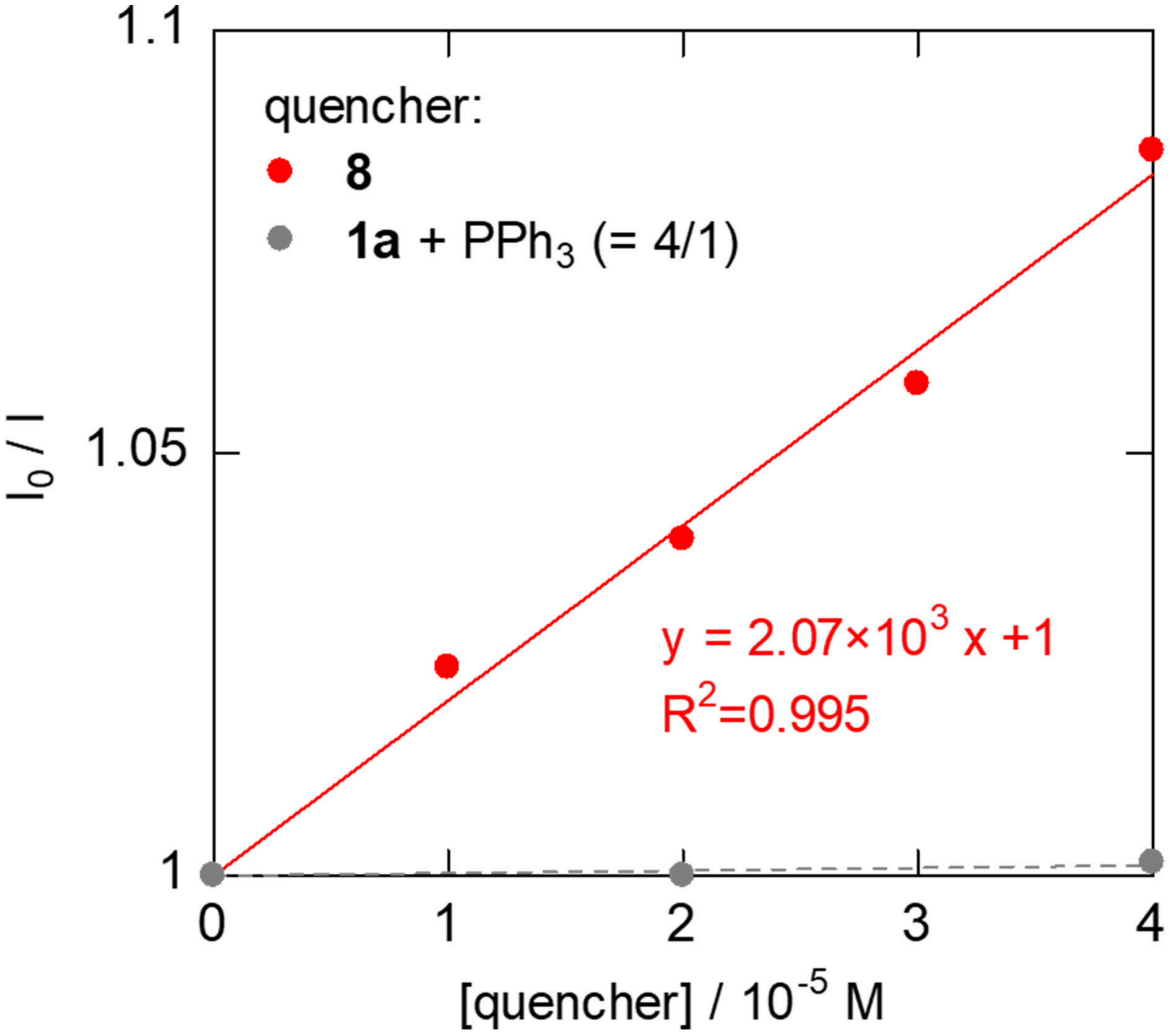
Luminescent quenching experiments of [Ru(bpy)_3_]^2+^ (1.0 × 10^−5^ M, deaerated DMF, r.t., λ_ex._ = 450 nm). **8** was generated *in situ* by the addition of 5 equiv. of **1a** to the DMF solution of **6**.

To investigate the carboxylation process with CO_2_, a DMA solution of a 1:1 mixture of *in-situ* generated **8** and PPh_3_ was exposed to the atmospheric pressure of CO_2_ under various conditions. The carboxylation did not proceed under dark even by heating, which was against our expectations based on the general reactivity of organorhodium(I) complexes with CO_2_ (Ukai et al., 2006; Mizuno et al., 2011; Suga et al., 2014; Kawashima et al., 2016). Quite interestingly, when the mixture was irradiated by visible-light for 30 min in the presence of 30 mol% of [Ru(bpy)_3_](PF_6_)_2_, **8** was successfully converted to the Rh(I) carboxylate complex, Rh(PPh_3_)_3_(η^1^-O_2_CCHCH_3_(4-CNC_6_H_4_)) (**9**), almost quantitatively (Figure 6ii). ^31^P{^1^H} NMR spectroscopy confirmed the clean formation of **9**: a pair of the doublet of doublet signals attributed to **8** completely disappeared with the PPh_3_ signal, and the doublet of doublet and doublet of triplet signals attributed to **9** appeared in 2: 1 ratio by visible-light irradiation (Figure 7). The control experiments demonstrated that CO_2_, [Ru(bpy)_3_](PF_6_)_2_ and visible-light were all essential for the carboxylation, suggesting that the nucleophilic addition of **8** to CO_2_ was facilitated by the photosensitization of [Ru(bpy)_3_]^2+^. The luminescence quenching experiment demonstrated that the excited state of [Ru(bpy)_3_]^2+^ was effectively quenched by **8** (Figure 8). The quenching constant was determined to be *K*_*q*_ = 2.07 × 10^3^, which was much larger than that by ^*i*^Pr_2_NEt (*K*_*q*_ = 1.56 × 10^2^, Supplementary Figure 3A). This result indicates that either photoinduced electron transfer or triplet-triplet energy transfer to **8** contributed to the quenching (Campagna et al., 2007; Arias-Rotondo and McCusker, 2016; Strieth-Kalthoff et al., 2018). However, the photoinduced electron transfer mechanism was unlikely in this case since (i) the carboxylation of **8** proceeded with a catalytic amount of [Ru(bpy)_3_](PF_6_)_2_ even in the absence of the sacrificial electron donor, and (ii) the cyclic voltammogram of **8** showed no significant redox peak within the window where the oxidative quenching of [Ru(bpy)_3_]^2+^ was possible. Therefore, the photoinduced triplet-triplet energy transfer from the excited [Ru(bpy)_3_]^2+^ to **8** was considered to be the most likely process in this carboxylation process. Although not very common, several examples on the photocatalytic organic transformations mediated by the triplet-triplet energy transfer were previously reported (Ikezawa et al., 1986; Osawa et al., 2001; Islangulov and Castellano, 2006; Lu and Yoon, 2012; Farney and Yoon, 2014).

**Figure 9:**
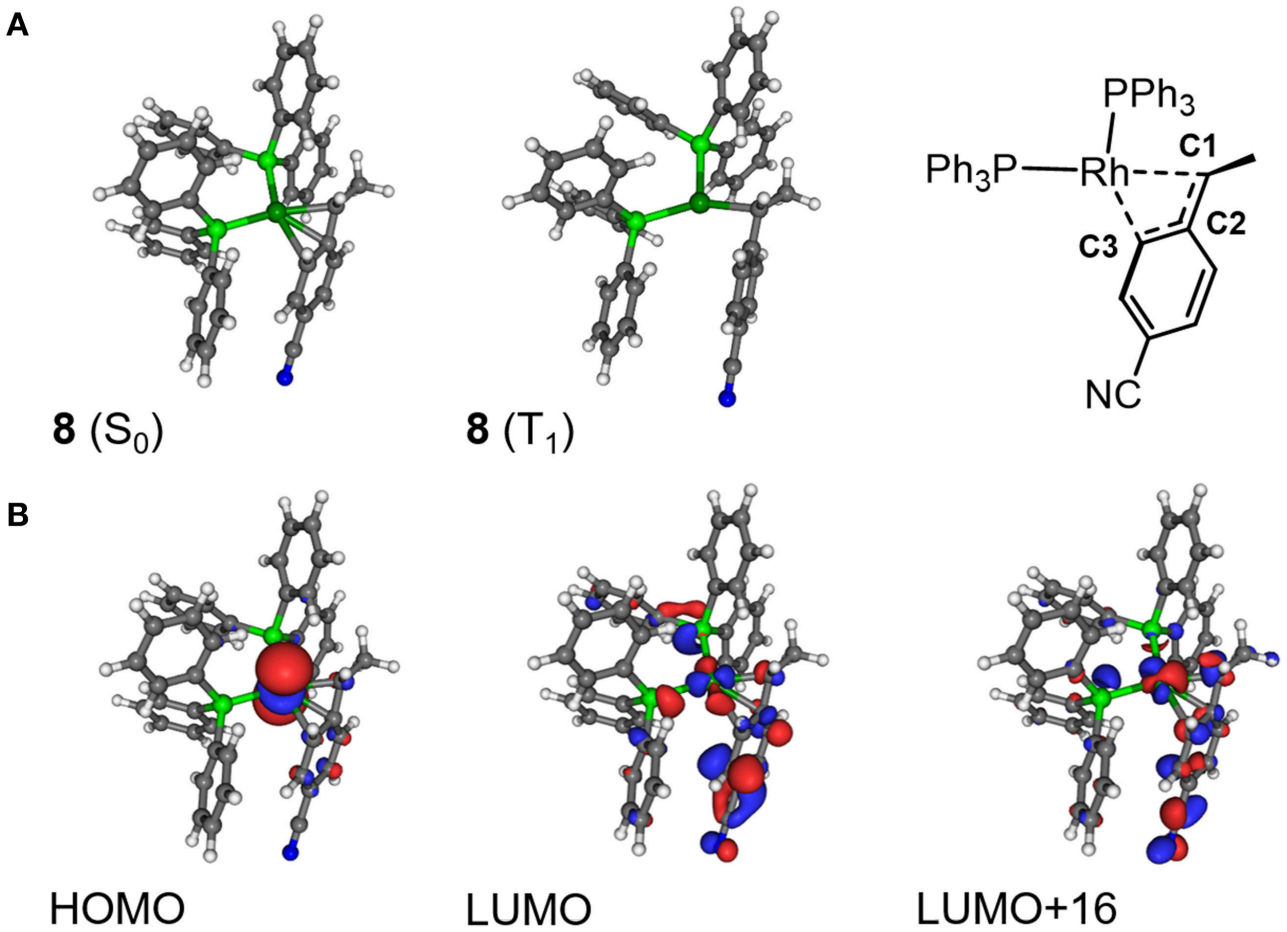
**(A)** Optimized structures of **8** in the ground (S_0_) state and lowest excited triplet (T_1_) state, and **(B)** the selected molecular orbitals of **8** (S_0_).

In order to investigate the detailed effect of the photoinduced energy transfer, the electronic structure analyses were performed in terms of the ground (S_0_) and the lowest excited triplet (T_1_) states of **8** based on DFT/TD-DFT methods. The calculated energy level of the T_1_ state of **8** (1.07 eV, based on the comparison between the S_0_ and T_1_ optimized geometries) was much lower than that of [Ru(bpy)_3_]^2+^ (2.17 eV), indicating that the triplet-triplet energy transfer from the excited [Ru(bpy)_3_]^2+^ to **8** was feasible. In terms of the optimized structures, a notable difference was found on the coordination manner of the benzyl ligand between the S_0_ and T_1_ geometries. In the S_0_ optimized structure, the η^3^-coodination of the benzyl ligand was represented by the similar three Rh-C distances, which coincided with the results of the ^1^H NMR observation (Figure 9A; Table 4). On the other hand, in the T_1_ optimized structure, while the Rh-C1 (benzyl carbon) distance remained unchanged, the Rh-C2/C3 distances significantly elongated compared with those of the S_0_ structure. These results indicated that the benzyl ligand changed its coordination-mode from η^3^-type (α-benzyl) to η^1^-type (σ-benzyl) in the T_1_ state. According to the analysis on the electronic transition characters, the T_1_ state was mainly contributed by the transitions of HOMO → LUMO (88%) and HOMO → LUMO+16 (4%) (Supplementary Table 2). The molecular orbital distribution indicated that LUMO and LUMO+16 mainly localized on the Rh (dπ) and benzyl ligand (π^*^) while the HOMO localized on the Rh (dσ) center (Figure 9B). As these LUMOs partially possessed the antibonding character on the Rh-C2/C3 bonds, the photoexcitation induced the dissociation of these Rh-C bonds, which resulted in the isomerization to the σ-benzyl species.

**Table 4:** Selected bond lengths and NBO natural charges in the optimized geometries of **8**(S_0_) and **8**(T_1_).

**State**	**S_**0**_**	**T_**1**_**
*l*(Rh-C1)/Å	2.184	2.176 (−0.008)
*l*(Rh-C2)/Å	2.225	2.510 (+0.285)
*l*(Rh-C3)/Å	2.295	3.189 (+0.894)
NBO Rh	−0.547	+0.158 (+0.705)
charge C1	−0.287	−0.412 (−0.125)

Concerning the acceleration of the carboxylation step, one possibility is the generation of the coordination site by taking σ-benzyl structure in the T_1_ state, which would promote the following carboxylation by facilitating coordination of CO_2_ to Rh center. Indeed, a similar thermal process has been proposed as a plausible mechanism for the carboxylation of organorhodium(I) complexes (Darensbourg et al., 1987). Another possibility is the direct nucleophilic addition of the benzyl carbon to CO_2_ in the T_1_ state. The NBO analysis demonstrated that the natural charge on the C1 atom significantly shifted to the negative side while that on the Rh atom shifted to the positive side in the T_1_ state. The increase of the electron density on the C1 atom in the T_1_ state would result in the acceleration of the nucleophilic addition to CO_2_. Therefore, the structural and/or electronic factors associated with the transition to the T_1_ state are thought to contribute to the carboxylation of **8**.

The effect of the photoactivation of the Rh(I) π-benzyl complex was also supported by the reactivity of the Rh(I) σ-alkyl complex with CO_2_. When a mixture of **6** and an excess amount of methyl acrylate (**1f**) was subjected to a CO_2_ atmosphere for 3 h even under dark, the quantitative formation of Rh(PPh_3_)_3_(η^1^-O_2_CCHCH_3_(CO_2_CH_3_)) (**10**) was indicated by ^31^P{^1^H} NMR spectroscopy. The carboxylation of **1f** was confirmed by the fact that the corresponding hydrocarboxylated product (**2f**) was obtained from the reaction mixture. This result indicated that the photosensitization by [Ru(bpy)_3_]^2+^ was not essential in this case. Thus, the major role of the excitation was thought to be the transformation from π-benzyl to σ-benzyl complexes to generate a coordination site and to make them more nucleophilic.

#### Addition of the Second Photosensitizer

The above mechanistic study revealed that a photosensitizer played two key roles in the hydrocarboxylation cycle: one is a “photoredox catalyst” to reduce the Rh(I) carboxylate species, and the other is a “triplet photosensitizer” to promote carboxylation of the Rh(I) benzyl species. With a single photosensitizer, the excited state of the photosensitizer was quenched by either a tertiary amine for the electron transfer or a Rh(I) benzyl species for the energy transfer, and these two processes competed during the reaction. Since the former was related to the rate-determining step when using ^*i*^Pr_2_NEt as a sacrificial electron donor, the incorporation of the second photosensitizer possessing suitable redox properties for the reductive quenching cycle was expected to facilitate the catalytic reaction.

On the basis of the idea, 2.0 mol% of a cyclometalated Ir(III) complex was added as a second photosensitizer to a mixture of **1b**, 3.5 mol% of **4**, 2.0 mol% of [Ru(bpy)_3_](PF_6_)_2_ and 4.0 equiv. of ^*i*^Pr_2_NEt, and the solution was irradiated under CO_2_ atmosphere at room temperature (Table 5). To excite both photosensitizers, a wide range of UV-visible-light (380–750 nm) was applied to the reactions. As expected, the addition of the second photosensitizer was found to be effective. For instance, when [Ir(ppy)_2_(dtbbpy)](PF_6_) was added, the reaction was completed after irradiation for only 6 h, and the yield of **2b** was increased more than five-fold compared to that of the reaction without the second photosensitizer (Table 5, entry 2). According to the redox properties of [Ir(ppy)_2_(dtbbpy)](PF_6_), the acceleration of the reaction was thought to be attributed mainly to the high reducing ability of the one-electron reduced species to promote the reduction process. The yield of **2b** further increased when incorporating [Ir(dF(CF_3_)ppy)_2_(dtbbpy)](PF_6_) as a second photosensitizer (Table 5, entry 3), and its concentration could be reduced to 1.0 mol% without lowering the yield (Table 5, entry 4). This result was assumed to be due to the high oxidizing ability of the excited state in addition to the sufficient reducing ability of the one-electron reduced species. The excited state of [Ir(dF(CF_3_)ppy)_2_(dtbbpy)](PF_6_) was found to be able to work as an energy transfer agent of **8** based on the luminescence quenching experiment (*K*_*q*_ = 2.76 × 10^4^, Supplementary Figure 3B). However, it is considered to contribute to the reaction mainly as an electron transfer agent under the catalytic conditions owing to the efficient quenching by the sacrificial electron donor. On the other hand, the addition of *fac*-Ir(ppy)_3_ resulted in only a small acceleration, which was probably attributable to the inferior oxidizing ability in the excited state (Table 5, entry 6). These results demonstrate that photosensitizers possessing both high oxidizing ability of the excited state and high reducing ability of the one-electron reduced species are advantageous as a second photosensitizer. The positive result on the addition of the two appropriate photosensitizers reflected the fact that the catalytic cycle was composed of the multiple photochemical processes, and the acceleration of the reduction process led to the enhancement of the catalytic activity when using ^*i*^Pr_2_NEt as a sacrificial electron donor.

**Table 5:** Screening of the reaction conditions of the hydrocarboxylation of 3,5-bis(trifluoromethyl)styrene with a second photosensitizer.

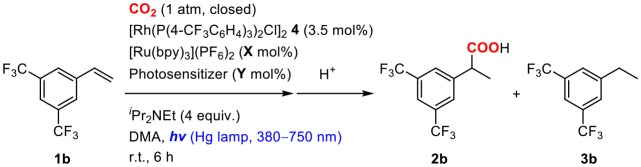					
**Entry**	**Photosensitizer**	**X/Y**	**Conv. /%**	**Yield /%**	
				**2b^a^**	**3b^b^**
1	None	2/0	37	7	9
2	[Ir(ppy)_2_(dtbbpy)](PF_6_)	2/2	>99	40	25
3	[Ir(dF(CF_3_)ppy)_2_(dtbbpy)](PF_6_)	2/2	>99	43	23
4	[Ir(dF(CF_3_)ppy)_2_(dtbbpy)](PF_6_)	2/1	>99	43	29
5	[Ir(dF(CF_3_)ppy)_2_(dtbbpy)](PF_6_)	1/2	90	24	16
6	*fac*-Ir(ppy)_3_	2/2	71	22	33
7^c^	None	2/0	74	45	19
8^c^	[Ir(dF(CF_3_)ppy)_2_(dtbbpy)](PF_6_)	2/2	53	23	7

a *NMR yield*.

b *GC yield*.

c *BI(OH)H (1.2 equiv.) was used instead of ^i^Pr_2_NEt (4.0 equiv.)*.

#### Rate-Determining Step in the Hydrocarboxylation With BI(OH)H

The previous experiments demonstrated that the rate-determining step of the catalytic cycle was the reduction process of the Rh(I) carboxylate species **9** when employing ^*i*^Pr_2_NEt as a sacrificial electron donor. In order to investigate the contribution of BI(OH)H to the catalytic cycle, the similar examination was carried out using BI(OH)H as a sacrificial electron donor instead of ^*i*^Pr_2_NEt. Interestingly, the resting-state was found to be Rh(I) π-benzyl intermediate **8** when a mixture of **1a**, catalytic amounts of **6** and [Ru(bpy)_3_](PF_6_)_2_, and 1.2 equiv. of BI(OH)H was irradiated by visible-light under CO_2_ atmosphere (Supplementary Figure 4). In that case, **9** was not detectable even after prolonged irradiation, indicating that the rate-determining step obviously altered from the reduction process to the carboxylation process by changing the sacrificial electron donor. This result was also supported by the fact that the acceleration of the reaction by the addition of a second photosensitizer was not observed in the case of the reaction using BI(OH)H as a sacrificial electron donor (Table 5, entry 8). These observations suggested that the use of BI(OH)H strongly accelerated the reductive quenching cycle of [Ru(bpy)_3_]^2+^ to promote the reduction process.

## Conclusion

In this study, the improved catalytic conditions of the visible-light driven hydrocarboxylation by Rh(I) and [Ru(bpy)_3_]^2+^ catalysts were explored, and the detailed reaction mechanism was investigated. On the basis of the stoichiometric reactions of the possible rhodium intermediates, the proposed catalytic cycle was confirmed to be composed of (i) the hydrometallation of alkenes by Rh(I) monohydride species, (ii) the photochemical carboxylation of the Rh(I) benzyl species with CO_2_, (iii) the photoinduced 2-electron, 2-proton transfers to the Rh(I) carboxylate species, and (iv) the base-assisted carboxylic acid elimination. One strategy for the enhancement of the catalytic reaction was to employ BI(OH)H possessing superior reducing ability as a sacrificial electron donor instead of ^*i*^Pr_2_NEt. It successfully improved the efficiency of the reaction, which had been major challenges in the previous catalytic conditions. The alteration of the resting-state by changing the sacrificial electron donor indicated that the addition of BI(OH)H significantly promoted the reduction process of the Rh(I) carboxylate species through the enhancement of the reductive quenching efficiency of [Ru(bpy)_3_]^2+^. Another strategy for the enhancement of the efficiency was to add the second photosensitizer in charge of the reductive quenching cycle. The acceleration of the catalytic reaction by the addition of the appropriate cyclometalated Ir(III) complex together with [Ru(bpy)_3_]^2+^ supported this hypothesis. These two effective strategies suggested that the promotion of the reduction processes was a key to enhance the catalytic activity in the present system. In addition to expand the versatility of the present hydrocarboxylation, this study would provide fundamental insights into the catalytic organic transformations by transition-metal/photoredox dual catalysis.

## Experimental

### General

All operations were carried out under an argon atmosphere unless otherwise noted. ^1^H, ^13^C, and ^31^P NMR spectra were recorded on Bruker DRX-500, JEOL ECZ-500, ECX-400, and ECS-400 spectrometers. ^31^P and ^19^F NMR chemical shifts were calibrated using external 85% H_3_PO_4_ (δ: 0.0 ppm) and neat C_6_F_6_ (δ: −164.9 ppm), respectively. IR spectra were recorded on an SC-100-VIR with an ATR PRO450-S accessory (JASCO Co., Ltd.). Emission spectra were recorded on an FP-6500 spectrofluorometer (JASCO Co., Ltd.). FAB-MS and FD-MS spectra were recorded on a JEOL JMS-700 spectrometer and a JMS-T100 spectrometer, respectively. Gas chromatography (GC-FID / TCD) was recorded on a Shimadzu GC-2010 spectrometer. Analytical thin-layer chromatography (TLC) was performed with a glass plate coated with silica gel (Wakogel B-5F). Visible-light irradiation was performed with a Relyon Twin LED Light (3W × 2, λ_irr._ = 425 ± 15 nm), and UV-visible-light irradiation was performed with an USHIO Optical Modulex OPM2-502XQ (500 W Xe lamp) with a super cold filter ZSC0750 (ASAHI Spectra Inc.).

THF, toluene, pentane and diethyl ether were purified by a solvent purification system by Glass Contour. Dehydrated dimethylacetamide (DMA) and dimethylformamide (DMF) were purchased from Kanto Chemical Co., Inc., degassed by argon bubbling and stored in a glovebox. Tertiary amines were distilled, degassed three times by freeze-pump-thaw method and stored under N_2_. Solvents for NMR measurements were dried over molecular sieves, degassed three times by freeze-pump-thaw method and stored under N_2_. All other solvents were distilled, degassed by argon bubbling and stored in a glovebox. CO_2_ and H_2_ gases were purchased from Taiyo Nippon Sanso Corporation. [Rh(coe)_2_Cl]_2_ (Van der Ent et al., 1990), P(4-CF_3_C_6_H_4_)_3_ (Suomalainen et al., 2001), Rh(PPh_3_)_2_(OAc) (**5**) (Grushin et al., 1995), [Rh(cod)(OAc)]_2_ (Chatt and Venanzi, 1957), Rh(PPh_3_)_3_H (**6**) (Annibale and Song, 2014), [Ru(bpy)_3_](PF_6_)_2_ (Damrauer et al., 1997), [Ru(dmbpy)_3_](PF_6_)_2_ (Damrauer et al., 1997), [Ru(bpz)_3_](PF_6_)_2_ (Schultz et al., 2015), Ir(ppy)_2_(dtbbpy)(PF_6_) (Tellis et al., 2014), [Ir(dF(CF_3_)ppy)_2_(dtbbpy)](PF_6_) (Slinker et al., 2004), *fac*-Ir(ppy)_3_ (Tamayo et al., 2003), and diisopropylethylammonium acetate (Anouti et al., 2008) were prepared according to the published methods. 4-Cyanostyrene (**1a**) was prepared by Wittig reaction of 4-cyanobenzaldehyde (Falk et al., 2013). Other chemicals were purchased and used as received.

### Photocatalytic Reactions

For screening conditions with alkenes (**1a**–**1g**), a DMA solution (0.6 mL) of an alkene (0.060 mmol), [Rh(P(4-CF_3_C_6_H_4_)_3_)_2_Cl]_2_ (**4**, 4.5 mg, 0.0021 mmol), photoredox catalyst(s), sacrificial electron donor and inorganic base (defined amounts) was prepared in a glass tube (φ 2.0 cm, 18 cm) under an argon atmosphere. Then the headspace gas was replaced by an atmospheric pressure of CO_2_, and the reaction vessel was put in a water bath placed at a distance of 10 mm from light sources. The mixture was irradiated with visible-light from blue LED lamp (λ_irr._ = 425 nm, two sockets) or UV-visible-light from Xe lamp (λ_irr._ = 380–800 nm) for defined time in the closed system. The product mixture was analyzed by ^1^H NMR and GC to determine the NMR yield of the hydrocarboxylated product (**2a**–**2g**) and the GC yield of the hydrogenated product (**3a**, **3b**), respectively (internal standard: 1,1,2,2-tetrachloroethane).

For isolation of the methyl esters of the hydrocarboxylated products (**2a**, **2b**, **2d**), a DMA solution (1.2 mL) of a styrene (0.12 mmol), **4** (9.0 mg, 0.0042 mmol), [Ru(bpy)_3_](PF_6_)_2_ (1.0 mg, 0.0012 mmol), BI(OH)H (58 mg, 0.24 mmol), and Cs_2_CO_3_ (47 mg, 0.14 mmol) was prepared in a glass tube (φ 2.0 cm, 18 cm), and irradiated with visible-light from blue LED lamp (λ_irr._ = 425 nm, three sockets) for defined time after replacement of the headspace gas by an atmospheric pressure of CO_2_. After irradiation, the reaction mixture was diluted with diethyl ether and extracted with H_2_O three times. The combined aqueous layer was acidified by 1N HCl aq., and then extracted with diethyl ether three times. The combined organic layer was dried over MgSO_4_, filtered and evaporated under reduced pressure to give the hydrocarboxylated product. Then, the product was dissolved in Et_2_O-MeOH, and TMSCHN_2_ (excess) was added at 0°C. The mixture was stirred at 0°C for 30 min and the solvent was removed under reduced pressure. The crude product was purified by preparative TLC (AcOEt/*n*-hexane = 1/5) to give the corresponding methyl-esterified product.

### Preparations of Rhodium Complexes and Their Stoichiometric Reactions

#### Preparation of [Rh(P(4-CF_3_C_6_H_4_)_3_)_2_Cl]_2_ (4)

A solution of P(4-CF_3_C_6_H_4_)_3_ (200 mg, 0.429 mmol) in toluene (2 mL) was added dropwise to a solution of [Rh(coe)_2_Cl]_2_ (77 mg, 0.107 mmol) in toluene (2 mL) and the mixture was stirred at room temperature overnight. After removal of solvent under reduced pressure, the crude product was dissolved in THF and then pentane was added to induce precipitation. The precipitates were collected to give the target product (218 mg, 0.102 mmol, 95% yield). ^1^H NMR (500 MHz, THF-*d*_8_, r.t., δ/ppm): δ 7.73–7.67 (m, 24 H, P*Ar*_3_), 7.39 (d, *J* = 8 Hz, 24 H, P*Ar*_3_). ^13^C{^1^H} NMR (125 MHz, r.t., THF-*d*_8_, δ/ppm): δ 139.3 (vt, *N* = 22 Hz, P*Ar*_3_), 135.7 (s, P*Ar*_3_), 132.1 (q, *J*_C−F_ = 33 Hz, P*Ar*_3_), 125.0 (s, P*Ar*_3_), 124.6 (q, *J*_C−F_ = 272 Hz, -*C*F_3_). ^31^P{^1^H} NMR (202 MHz, THF-*d*_8_, r.t., δ/ppm): δ 53.4 (d, *J* = 194 Hz). ^19^F NMR (471 MHz, THF-*d*_8_, r.t., δ/ppm): δ −60.6 (s). ESI-MS: *m/z* = 1035 [M/2 − Cl]^+^. Anal. Found (calcd for C_84_H_48_Cl_2_F_36_P_4_Rh_2_): C, 46.94 (47.11); H, 2.28 (2.26).

#### Preparation of Rh(PCy_3_)_2_(OAc) (5′)

[Rh(cod)(OAc)]_2_ (60 mg, 0.111 mmol) and PCy_3_ (125 mg, 0.444 mmol) were suspended in DMA (4 mL), and the mixture was irradiated with UV-visible-light (500 W Xe lamp, λ_irr._ = 380–800 nm) at room temperature with vigorous stirring for 30 h. The precipitate was filtered, washed with DMA and cold pentane (−35°C), and then recrystallized from a minimum volume of pentane at −35°C to yield the target compound (98 mg, 0.135 mmol, 61%). IR (KBr): ν(OCO_as_) = 1,528, ν(OCO_sym_) = 1,445 cm^−1^. ^1^H NMR (500 MHz, C_6_D_6_, r.t., δ/ppm): δ 2.35 – 1.24 (m, 69 H, P*Cy*_3_ and O_2_CC*H*_3_). ^13^C{^1^H} NMR (125 MHz, r.t., C_6_D_6_, δ/ppm): δ 188.2 (s, O_2_*C*CH_3_), 35.9 (vt, *N* = 10 Hz, P*Cy*_3_), 31.1 (s, P*Cy*_3_), 28.4 (vt, *N* = 5 Hz, P*Cy*_3_), 27.3 (s, P*Cy*_3_), 24.9 (s, O_2_C*C*H_3_). ^31^P{^1^H} NMR (202 MHz, C_6_D_6_, r.t., δ/ppm): δ 59.1 (d, *J*_P−Rh_ = 198 Hz). HR-MS (FAB): *m/z* = 722.3817 [M]^+^ (calcd for [C_38_H_69_O_2_P_2_Rh]^+^: 722.3828).

#### Preparation of an Authentic Sample of Rh(PCy_3_)_2_(OAc)(H)_2_ (7′)

[Rh(cod)(OAc)]_2_ (60 mg, 0.111 mmol) and PCy_3_ (125 mg, 0.444 mmol) were dissolved in THF (3 mL), and the mixture was stirred under H_2_ (1 atm) at room temperature overnight. After removal of solvent, the crude product was dissolved in toluene and filtered through Celite®. The resultant solid after evaporation was washed with cold diethyl ether (−35°C) to give the target compound (113 mg, 0.156 mmol, 70%). IR (KBr): ν(RhH) = 2,143, ν(OCO_as_) = 1,551, ν(OCO_sym_) = 1,436 cm^−1^. ^1^H NMR (500 MHz, C_6_D_6_, r.t., δ/ppm): δ 2.20 – 1.22 (m, 69 H, P*Cy*_3_ and O_2_CC*H*_3_), −23.7 (dt, *J*_H−Rh_ = 24, *J*_H−P_ = 15 Hz, 2 H, Rh-*H*) ^13^C{^1^H} NMR (125 MHz, r.t., C_6_D_6_, δ/ppm): δ 180.2 (s, O_2_*C*CH_3_), 35.8 (vt, *J*_C−P_ = 10 Hz, P*Cy*_3_), 30.5 (s, P*Cy*_3_), 28.3 (vt, *N* = 5 Hz, P*Cy*_3_), 27.1 (s, P*Cy*_3_), 24.8 (s, O_2_C*C*H_3_). ^31^P{^1^H} NMR (202 MHz, C_6_D_6_, r.t., δ/ppm): δ 51.1 (d, *J*_P−Rh_ = 115 Hz). HR-MS (FD): *m/z* = 724.3975 (calcd for [C_38_H_71_O_2_P_2_Rh]^+^: 724.3984).

#### Preparation of Rh(PPh_3_)_2_(η^3^-CHCH_3_(4-CNC_6_H_4_)) (8)

To a solution of Rh(PPh_3_)_3_H (**6**) (5.4 mg, 0.0060 mmol) in THF-*d*_8_ (0.6 mL) in a J. Young NMR tube was added 4-cyanostyrene (**1a**) (1.6 μL, 0.012 mmol) at room temperature. Rh(PPh_3_)_2_(η^3^-CHCH_3_(4-CNC_6_H_4_)) (**8**) formed almost quantitatively. ^1^H NMR (500 MHz, THF-*d*_8_, −10°C, δ/ppm): δ 7.47 – 7.00 (m, 30 H, P*Ph*_3_), 6.81 (brd, *J* = 8 Hz, 1 H, *Ar*), 6.57 (brd, *J* = 7 Hz, 1 H, *Ar*), 6.10 (brd, *J* = 8 Hz, 1 H, *Ar*), 4.89 (brd, *J* = 7 Hz, 1 H, *Ar*), 2.47 – 2.40 (m, 1 H, -C*H*CH_3_), 0.92 – 0.87 (m, 3 H, -CHC*H*_3_). ^31^P{^1^H} NMR (202 MHz, THF-*d*_8_, −10°C, δ/ppm): δ 46.2 (dd, *J*_P−Rh_ = 263 Hz, *J*_P−P_ = 31 Hz), 39.6 (dd, *J*_P−Rh_ = 178 Hz, *J*_P−P_ = 31 Hz). HR-MS (FAB): *m/z* = 757.1539 (calcd for [C_45_H_38_NP_2_Rh]^+^: 757.1535).

#### Preparation of Rh(PPh_3_)_3_(η^1^-O_2_CCHCH_3_(4-CNC_6_H_4_)) (9)

To a solution of Rh(PPh_3_)_3_H (**6**) (60 mg, 0.090 mmol) in toluene (3 mL) was added dropwise a solution 2-(4-cyanophenyl)propionic acid (16 mg, 0.090 mmol) in toluene (2 mL), and the mixture was stirred at room temperature for 4 h. After removal of solvent under reduced pressure, the crude product was dissolved in toluene and then pentane was added to induce precipitation. The precipitates were collected to give the target product as a 9: 1 mixture with Rh(PPh_3_)_2_(η^2^-O_2_CCHCH_3_(4-CNC_6_H_4_)) which was formed by dissociation of PPh_3_ from **9** (59 mg). **9** IR (KBr): ν(CN) = 2,224, ν(OCO_as_) = 1,604, ν(OCO_sym_) = 1,342 cm^−1^. ^1^H NMR (500 MHz, THF-*d*_8_, −60°C, δ/ppm): δ 7.56 – 6.79 (m, 47 H, P*Ph*_3_, O_2_CCHCH_3_*Ar*), 6.30 (brd, *J* = 8 Hz, 2 H, O_2_CCHCH_3_*Ar*), 1.65 (brq, *J* = 7 Hz, 1 H, O_2_CC*H*CH_3_Ar), 0.25 (brd, *J* = 7 Hz, 3 H, O_2_CCHC*H*_3_Ar). ^13^C{^1^H} NMR (125 MHz, r.t., THF-*d*_8_, −30°C, δ/ppm): δ 176.6 (s, O_2_*C*CHCH_3_Ar), 150.8 (s, O_2_CCHCH_3_*Ar*), 135.7 (vt, *N* = 6 Hz, P*Ph*_3_), 131.1 (s, O_2_CCHCH_3_*Ar*), 129.6 (s, O_2_CCHCH_3_*Ar*), 129.2 (s, P*Ph*_3_), 127.8 (s, P*Ph*_3_), 127.5 (s, P*Ph*_3_), 127.4 (s, P*Ph*_3_), 119.8 (s, -*C*N), 108.9 (s, O_2_CCHCH_3_*Ar*), 47.8 (s, O_2_C*C*HCH_3_Ar), 18.1 (s, O_2_CCH*C*H_3_Ar). ^31^P{^1^H} NMR (202 MHz, THF-*d*_8_, −30°C, δ/ppm): δ 51.5 (dt, *J*_P−Rh_ = 174 Hz, *J*_P−P_ = 41 Hz), 34.9 (dd, *J*_P−Rh_ = 153 Hz, *J*_P−P_ = 41 Hz). HR-MS (FAB): *m/z* = 801.1453 [M−(PPh_3_)]^+^ (calcd for [C_46_H_38_NO_2_P_2_Rh]^+^: 801.1433). Rh(PPh_3_)_2_(η^2^-O_2_CCHCH_3_(4-CNC_6_H_4_)) ^31^P{^1^H} NMR (202 MHz, THF-*d*_8_, −30°C, δ / ppm): δ 57.6 (brd, *J*_P−Rh_ = 210 Hz).

#### Redox-Photosensitized Reaction of Rh(PPh_3_)_2_(OAc) (5)

Rh(PPh_3_)_2_(OAc) (**5**) (2.7 mg, 0.0040 mmol), [Ru(bpy)_3_](PF_6_)_2_ (1.0 mg, 0.0012 mmol), PPh_3_ (1.5 mg, 0.0060 mmol), ^*i*^Pr_2_NEt (41 μL, 0.24 mmol) and DMA (0.6 mL) were added in a glass tube with a magnetic stirrer. The mixture was irradiated with visible-light (λ_irr._ = 425 nm) at room temperature for 6 h. The solvent was removed under reduced pressure, and the resulting solid was analyzed by ^1^H and ^31^P NMR spectroscopies (THF-*d*_8_, internal standard: mesitylene). The Rh(I) monohydride species corresponding to Rh(PPh_3_)_3_H (**6**) formed in 57% yield (based on the Rh-hydride signal) and ca. 30% of the starting material **5** remained in the product mixture. **6** was highly fluxional in the reaction solution at room temperature in the presence of triphenylphosphine. ^1^H NMR (500 MHz, THF-*d*_8_, δ/ppm, r.t.): −8.45 (brd, *J*_H−Rh_ = 13 Hz, Rh-*H*). ^31^P NMR (202 MHz, THF-*d*_8_, δ/ppm, r.t.): 41 (br). The intensity of the Rh-hydride signal was significantly increased at room temperature when Rh(PPh_3_)_3_H (**6**) synthesized alternatively was added to the reaction mixture, also supporting the formation of **6**. In addition, when the solution was cooled to −90°C, the signals attributed to Rh(PPh_3_)_4_H were observed instead, indicating **6** was converted to Rh(PPh_3_)_4_H at low temperature. ^1^H NMR (500 MHz, THF-*d*_8_, δ/ppm, −90°C): −13.5 (dq *J*_H−Rh_ = 118, *J*_H−P_ = 15 Hz, Rh-*H*). ^31^P NMR (202 MHz, THF-*d*_8_, δ/ppm, −90°C): 33.4 (dm, *J*_P−Rh_ = 112), 30.2 (dd, *J*_P−Rh_ = 162, *J*_P−P_ = 32 Hz). The spectrosopic data was analogous to the values reported previously (Dewhirst et al., 1968; Strauss and Shriver, 1978).

#### Redox-Photosensitized Reaction of Rh(PCy_3_)_2_(OAc) (5′)

Rh(PCy_3_)_2_(OAc) (**5′**) (2.9 mg, 0.0040 mmol), [Ru(bpy)_3_](PF_6_)_2_ (1.0 mg, 0.0012 mmol), ^*i*^Pr_2_NEt (41 μL, 0.24 mmol) and DMA (0.6 mL) were added in a glass tube with a magnetic stirrer. The mixture was irradiated with visible-light (λ_irr._ = 425 nm) at room temperature for 12 h. The solvent was removed under reduced pressure, and the resulting solid was analyzed by ^1^H and ^31^P NMR spectroscopies (C_6_D_6_, internal standard: mesitylene). Rh(PCy_3_)(OAc)(H)_2_ (**7′**) was formed in 94% yield (based on the rhodium hydride signal). The spectroscopic feature well agreed with the complex **7′** synthesized alternatively (*vide supra*). No other Rh hydride signal was observed even by addition of 1 equiv. of PCy_3_ to the reaction mixture.

#### Reaction of Rh(PPh_3_)_2_(OAc)(H)_2_ (7) and *^*i*^*Pr_2_NEt

A solution of Rh(PPh_3_)_2_(OAc) (**5**) (2.7 mg, 0.0040 mmol) in C_6_D_6_ (0.6 mL) was put in a J. Young NMR tube under hydrogen atmosphere at room temperature. After 2 h, Rh(PPh_3_)_2_(OAc)(H)_2_ (**7**) formed *in situ*. ^1^H NMR (500 MHz, C_6_D_6_, δ/ppm, r.t.): 7.86 – 7.00 (m, P*Ph*_3_), −20.8 (dt, *J*_H−Rh_ = 22 Hz, *J*_H−P_ = 17 Hz, Rh-*H*). ^31^P{^1^H} NMR (202 MHz, C_6_D_6_, δ/ppm, r.t.): 41.4 (d, *J*_P−Rh_ = 121 Hz). Then, the reaction solution was degassed by freeze-pump-thaw method 3 times to remove the hydrogen gas. Addition of 1 equiv. of PPh_3_ (1.5 mg, 0.0060 mmol) and 80 equiv. of ^*i*^Pr_2_NEt (41 μL, 0.24 mmol) gave Rh(PPh_3_)_3_H (**6**) in 58% yield (based on the rhodium hydride signal). Ca. 20% of **7** remained in the reaction mixture. The spectroscopic data of **6** was identical with that of the sample prepared by the photosensitizing reaction of Rh(PPh_3_)_2_(OAc) (**5**). The same reaction was also performed in DMA. In this case, **7** converted fully after addition of PPh_3_ and ^*i*^Pr_2_NEt, and **6** was obtained as a major product.

#### Reaction of Rh(PPh_3_)_3_H (6) and [*^*i*^*Pr_2_NHEt]^+^[CH_3_COO]^−^

To a solution of Rh(PPh_3_)_3_H (**6**) (3.6 mg, 0.0040 mmol) in C_6_D_6_ (0.6 mL) was added diisopropylethylammonium acetate (1.1 mg, 0.0060 mmol) and the mixture was stirred at room temperature for 10 min. Rh(PPh_3_)_2_(OAc)(H)_2_ (**7**) formed quantitatively. The spectroscopic data of the product in C_6_D_6_ was identical with those of the sample prepared by the hydrogenation of Rh(PPh_3_)_2_(OAc) (**5**).

#### Redox-Photosensitized Reaction of Rh(PPh_3_)_3_Cl

Rh(PPh_3_)_3_Cl (3.7 mg, 0.0040 mmol), [Ru(bpy)_3_](PF_6_)_2_ (1.0 mg, 0.0012 mmol), ^*i*^Pr_2_NEt (41 μL, 0.24 mmol) and DMF-*d*_7_ (0.6 mL) were added in a J. Young NMR tube. The mixture was irradiated with visible-light (λ_irr._ = 425 nm) at room temperature for 6 h. Rh(PPh_3_)_3_H (**6**) formed in 52% yield (based on the Rh-hydride signal) and ca. 40% of the starting material remained in the reaction mixture. ^1^H NMR (500 MHz, δ/ppm, −50°C): −8.48 (ddt, *J*_H−Rh_ = 101 Hz, *J*_H−P_ = 18 Hz, *J*_H−P_ = 15 Hz, Rh-*H*). ^31^P NMR (202 MHz, δ/ppm, −50°C): 43.7 (dd, *J*_P−Rh_ = 170 Hz, *J*_P−P_ = 25 Hz), 38.9 (dm, *J*_P−Rh_ = 149 Hz). The spectrosopic data was analogous to the values reported previously (Dewhirst et al., 1968; Strauss and Shriver, 1978).

#### Reaction of Rh(PPh_3_)_3_H (6) With 4-Cyanostyrene (1a) and CO_2_

To a solution of Rh(PPh_3_)_3_H (**6**) (3.6 mg, 0.0040 mmol) in DMA (0.6 mL) was added 4-cyanostyrene (**1a**) (2.6 μL, 0.020 mmol) in a J. Young NMR tube at room temperature. Rh(PPh_3_)_2_(η^3^-CHCH_3_(4-CNC_6_H_4_)) (**8**) was generated *in situ* almost quantitatively. Then, [Ru(bpy)_3_](PF_6_)_2_ (1.0 mg, 0.0012 mmol) was added, and the mixture was irradiated with visible-light (λ_irr._ = 425 nm) under a CO_2_ atmosphere (1 atm) at room temperature for 30 min. Rh(PPh_3_)_3_(η^1^-O_2_CCHCH_3_(4-CNC_6_H_4_)) (**9**) was formed almost quantitatively, as the spectroscopic data well agreed with the complex synthesized alternatively.

#### Reaction of Rh(PPh_3_)_3_H (6) With Methyl Acrylate (1f) and CO_2_

To a solution of Rh(PPh_3_)_3_H (**6**) (3.6 mg, 0.0040 mmol) and [Ru(bpy)_3_](PF_6_)_2_ (1.0 mg, 0.0012 mmol) in DMA (0.6 mL) was added methyl acrylate (**1f**) (1.8 μL, 0.020 mmol) in a J. Young NMR tube at room temperature. The mixture was exposed to a CO_2_ atmosphere (1 atm) under dark at room temperature for 3 h. The observed ^31^P{^1^H} NMR spectral feature was analogous to that of **9**, indicating the formation of Rh(PPh_3_)_3_(η^1^-O_2_CCHCH_3_(CO_2_CH_3_)) (**10**). ^31^P{^1^H} NMR (162 MHz, DMA, −15°C, δ/ppm): δ 50.6 (dt, *J*_P−Rh_ = 174 Hz, *J*_P−P_ = 44 Hz), 33.5 (dd, *J*_P−Rh_ = 153 Hz, *J*_P−P_ = 44 Hz). The reaction solution was treated with NaHCO_3_ aq., and then acidified with 1N HCl aq. The organic layer was extracted with diethyl ether three times, dried over MgSO_4_, filtered and evaporated under reduced pressure to give methyl methylmalonate in 54% yield (based on **6**).

### Observation of the Reaction Intermediates Under Catalytic Conditions

Rh(PPh_3_)_3_H (**6**) (3.6 mg, 0.0040 mmol), [Ru(bpy)_3_](PF_6_)_2_ (1.0 mg, 0.0012 mmol), BI(OH)H (17.3 mg, 0.072 mmol), 4-cyanostyrene (**1a**) (7.7 μL, 0.060 mmol) and DMA (0.6 mL) were added in a J. Young NMR tube. The mixture was irradiated with visible-light (λ_irr._ = 425 nm) under CO_2_ atmosphere (1 atm) at room temperature for 30 min. The ^31^P{^1^H} NMR spectra were observed at −15°C before and after irradiation, and only Rh(PPh_3_)_2_(η^3^-CHCH_3_(4-CNC_6_H_4_)) (**8**) was observed as a resting-state in both spectra.

### Theoretical Study

Theoretical calculations were performed at the DFT level with the Gaussian 09 package. The geometry optimizations were performed using the mPW1PW91 functional (Adamo and Barone, 1998). The LanL2DZ basis set was used for all atoms and extended by a polarization function (except for H) (Dunning and Hay, 1976; Wadt and Hay, 1985a,b). To address solvation effects, the conductor-like polarizable continuum model (CPCM, N,N-Dimethylacetamide) (Tomasi et al., 2005) was used for the ground and excited states. For validation, vibrational frequencies were calculated for the ground and excited states. The orbital plots as well as the graphical representations were performed using Molekel (Varetto, 2009). Natural bond orbital (NBO) analysis was used to predict and interpret the computational results (Glendening et al., 2001). Total ZPE energies and cartesian coordinates of computed structures are given in Supplementary Table 3.

## Data Availability

All datasets generated for this study are included in the manuscript and/or the Supplementary Files.

## Author Contributions

NN and KM performed the experiments. KS instructed the experiments of NN. KM performed the theoretical calculations. NI and JT supervised the project. KM and NI wrote the paper.

### Conflict of Interest Statement

The authors declare that the research was conducted in the absence of any commercial or financial relationships that could be construed as a potential conflict of interest. The reviewer MA declared a shared affiliation, with no collaboration, with the authors NN, KS, JT, NI, to the handling editor at the time of review.

## Abbreviations

bpy: 2,2′-bipyridyl
dtbbpy: 4,4′-di-*tert*-butyl-2,2′-bipyridyl
bpz: 2,2′-bipyrazine
ppy: 2-phenylpyridiine
dF(CF_3_)ppy: 3,5-difluoro-2-[5-(trifluoromethyl)-2-pyridinyl]phenyl
SCE: saturated calomel electrode
BIH: 1,3-dimethyl-2-phenyl-2,3-dihydro-1*H*-benzo[*d*]imidazole
BI(OH)H: 1,3-dimethyl-2-(*o*-hydroxyphenyl)-2,3-dihydro-1*H*-benzo[*d*]imidazole
SED: sacrificial electron donor
PC: photoredox catalyst
SCE: saturated calomel electrode
vt, virtual coupling. For the abbreviations of the rhodium complexes: see Supplementary Table 1.
