# Supplementary material for: Improved Conditions for the Visible-Light Driven Hydrocarboxylation by Rh(I) and Photoredox Dual Catalysts Based on the Mechanistic Analyses
Source: Front Chem. 2019 May 22;7:371. doi: 10.3389/fchem.2019.00371 (PMC6558419; doi:10.3389/fchem.2019.00371)
Supplement: Supplementary file 1 [file Data_Sheet_1.PDF]

frontiers

Supplementary Material

Improved Conditions for the Visible-light Driven Hydrocarboxylation by Rh(I) and Photoredox Dual Catalysts based on the Mechanistic Analyses

*Kei Murata, Nobutsugu Numasawa, Katsuya Shimomaki, Jun Takaya, and Nobuharu Iwasawa**

Department of Chemistry, Tokyo Institute of Technology, O-Okayama, Meguro-ku, Tokyo 152-8551, Japan

Contents	Figure, Table	Page
**1. Abbreviations**		
Abbreviations of the Rh Complexes	Supplementary Table 1	S2
**2. Experimental Results**		
Photochemical Generations of the Rhodium Hydrides	Supplementary Figure 1	S3
	Supplementary Figure 2	S3
Luminescence Quenching Experiment	Supplementary Figure 3	S4
Observation of the Reaction Intermediates under Catalytic Conditions	Supplementary Figure 4	S5
**3. Theoretical Calculations**		
Energies and Compositions of the Calculated Triplet Excited States	Supplementary Table 2	S6
Cartesian Coordinates and ZPE Corrected Total Energy of the Optimized Structures	Supplementary Table 3	S7

1 Abbreviations

Supplementary Table 1. Abbreviations of the Rh Complexes.

Name	Simplified structure	Abbrev.	Specific structure	Abbrev.
Rh(I) monohydride	L_nRh^I—H	**A**	$(PPh_3)_3Rh$—H	**6**
Rh(I) benzyl	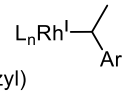 (σ-benzyl)	**B**	–	–
	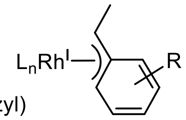 (π-benzyl)	**B'**	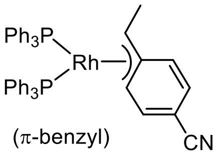 (π-benzyl)	**8**
Rh(I) carboxylate	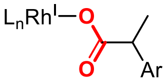	**C**	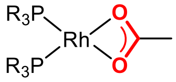	R = Ph **5**<br>Cy **5'**
			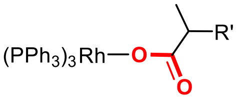	R' = 4-CNC_6H_4 **9**<br>CO_2Me **10**
Rh(III) dihydride carboxylate	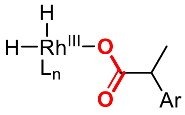	**D**	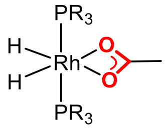	R = Ph **7**<br>Cy **7'**

2 Experimental Results

Photochemical Generations of the Rhodium Hydrides

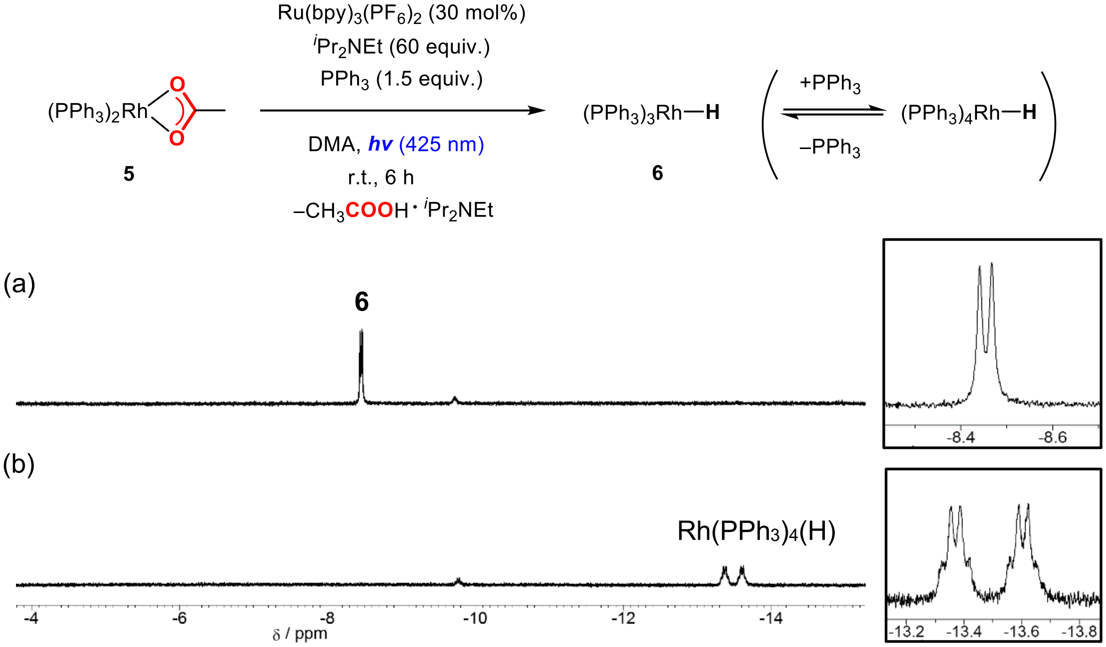

Supplementary Figure 1. $^{31}P\{^{1}H\}$ NMR spectra of the redox-photosensitized reaction of $Rh(PPh_3)_2(OAc)$ (**5**) observed at (a) r.t. and (b) -90 °C (500 MHz, THF-d_8, Rh hydride region).

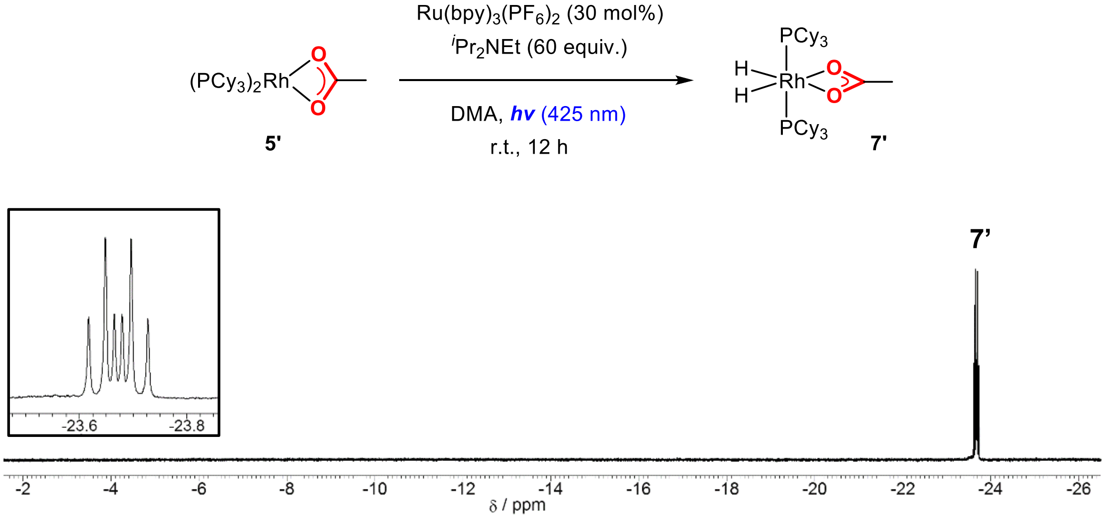

Supplementary Figure 2. $^{31}P\{^{1}H\}$ NMR spectrum of the redox-photosensitized reaction of $Rh(PCy_3)_2(OAc)$ (**5'**) observed at r.t. (500 MHz, THF-d_8, Rh hydride region).

Luminescence Quenching Experiment

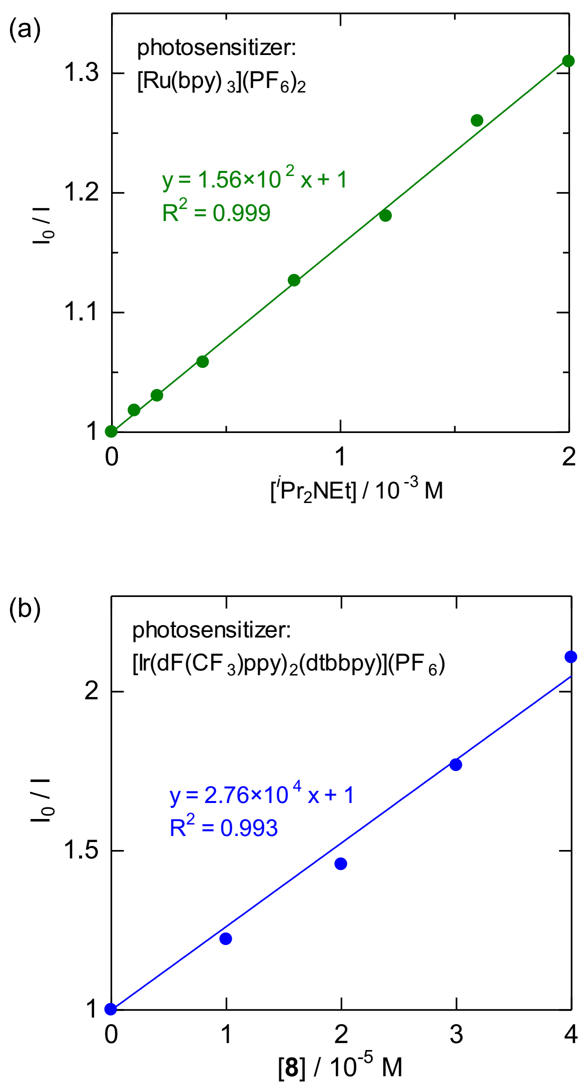

Supplementary Figure 3. Stern-Volmer plots of (a) $[Ru(bpy)_3](PF_6)_2$ (1.0×10^{-5} M, $\lambda_{ex.}$ = 450 nm) with $^{i}Pr_2NEt$ and (b) $Ir[dF(CF_3)ppy]_2(dtbbpy)](PF_6)$ (1.0×10^{-5} M, $\lambda_{ex.}$ = 380 nm) with **8** generated *in situ* in deaerated DMF at r.t..

Observation of the Reaction Intermediates under Catalytic Conditions

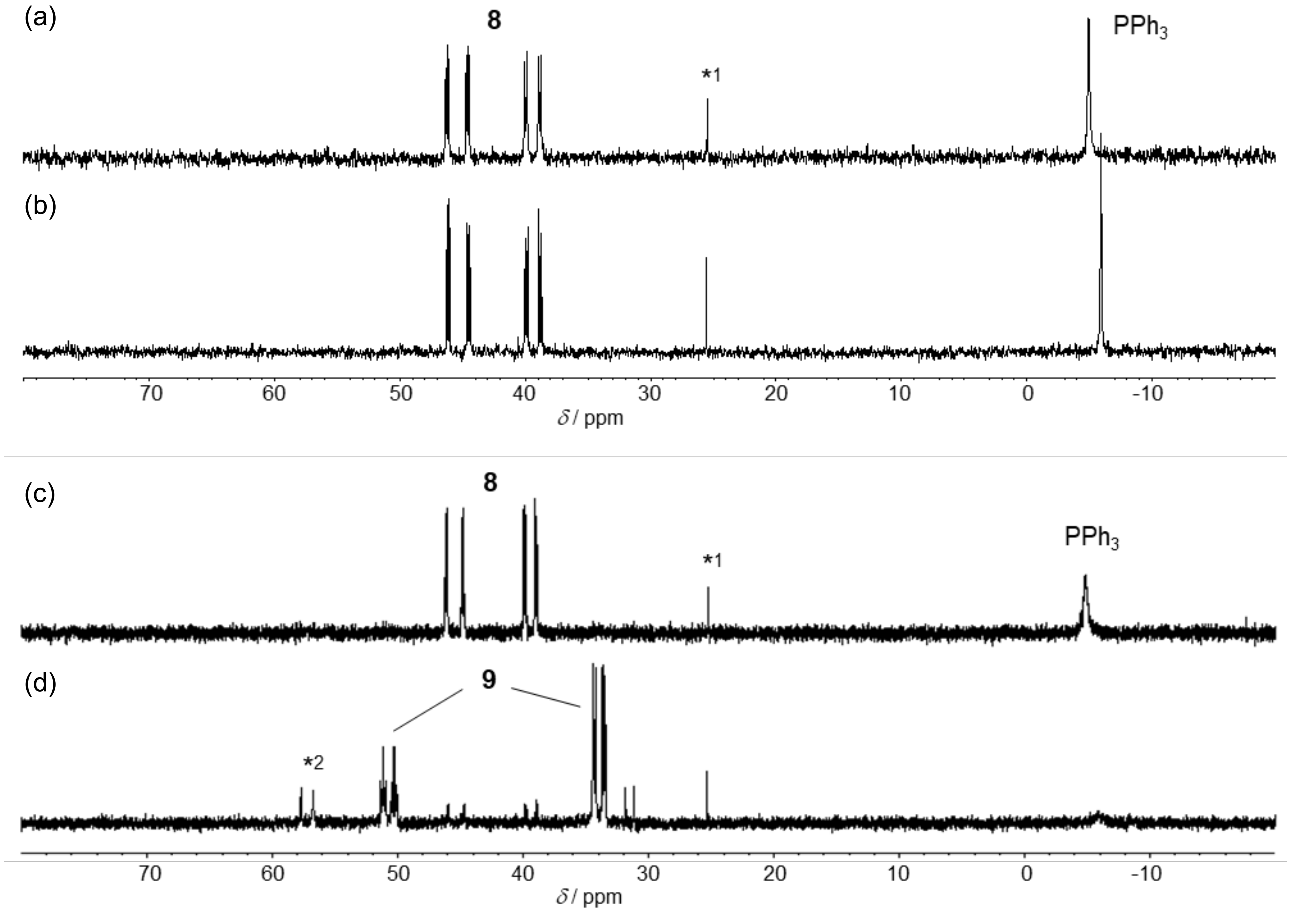

Supplementary Figure 4. $^{31}P\{^{1}H\}$ NMR spectra of the reaction with BI(OH)H (a) under dark and (b) visible-light irradiation for 30 min (500 MHz, DMA, -15 °C). The bottoms are $^{31}P\{^{1}H\}$ NMR spectra of the reaction with $^{i}Pr_2NEt$ (c) under dark and (d) visible-light irradiation for 30 min for comparison (Conditions: $Rh(PPh_3)_3H$ (**6**) (3.6 mg, 0.0040 mmol), $[Ru(bpy)_3](PF_6)_2$ (1.0 mg, 0.0012 mmol), $^{i}Pr_2NEt$ (41 µL, 0.24 mmol), 4-cyanostyrene (**1a**) (2.6 µL, 0.020 mmol), DMA (0.6 mL), CO_2 (1 atm), visible light ($\lambda_{irr.}$ = 425 nm), r.t.). ^{*1}(O=)PPh_3 ^{*2} $Rh(PPh_3)_2(\eta^2\text{-}O_2CCHCH_3(4\text{-}CNC_6H_4))$

3 Theoretical Calculations

Supplementary Table 2. Energies and Compositions of the Calculated Excited Triplet States of **8** (Calculated by TD-DFT Based on the S_0 Optimized Geometry, Transition Percentage > 3%)

State	*E* [eV]	*E* [10^4 cm^{-1}]	Major Contributions [%]	
T_1	1.85	1.49	88	HOMO → LUMO
			4	HOMO → LUMO+16
T_2	2.19	1.77	87	HOMO-1 → LUMO

Supplementary Table 3. Cartesian coordinates and ZPE corrected total energy of the optimized structures of **8**(S_0) and **8**(T_1).

8(S_0) E_{tot} = -1914.7863 a.u.

Rh	0.097049	-0.749110	-0.838960
P	1.385644	1.009860	-0.020756
P	-1.960485	-0.204368	-0.014757
C	-0.657622	-2.395620	-2.059530
C	1.457638	1.210692	1.820019
C	1.533825	0.019335	2.563394
H	1.503890	-0.939428	2.044258
C	1.646975	0.049421	3.956466
H	1.712591	-0.883850	4.513215
C	1.666651	1.276645	4.630767
H	1.747711	1.304848	5.716070
C	1.570696	2.466525	3.900700
H	1.572218	3.425107	4.417093
C	1.471032	2.435102	2.504225
H	1.384077	3.372838	1.961720
C	1.032896	2.684124	-0.738403
C	-0.028466	2.814117	-1.644743
H	-0.642850	1.942684	-1.870471
C	-0.285600	4.036855	-2.277163
H	-1.111679	4.117589	-2.981769
C	0.520131	5.147351	-2.006303
H	0.322407	6.100243	-2.494438
C	1.596427	5.023134	-1.116767
H	2.240106	5.877672	-0.915040
C	1.857811	3.798428	-0.496965
H	2.725824	3.708982	0.153861
C	3.195662	0.916328	-0.453920
C	4.193681	0.605252	0.479793
H	3.940866	0.463165	1.528148
C	5.530440	0.477467	0.077610
H	6.292337	0.235913	0.817074
C	5.886070	0.661909	-1.261889
H	6.924513	0.563618	-1.573507
C	4.893669	0.979228	-2.200653
H	5.157845	1.129413	-3.246215
C	3.561349	1.102338	-1.799986
H	2.802605	1.346417	-2.543134
C	-2.219963	1.097742	1.297628
C	-2.310773	0.765989	2.658978
H	-2.245088	-0.271667	2.977472
C	-2.511323	1.757502	3.626837
H	-2.588178	1.474587	4.675549
C	-2.619590	3.100567	3.251048
H	-2.782413	3.870999	4.002897
C	-2.521421	3.442948	1.896814
H	-2.607761	4.483304	1.587177
C	-2.321636	2.451554	0.931673
H	-2.270336	2.742934	-0.114732
C	-3.236633	0.331003	-1.254226
C	-2.926754	0.237573	-2.619009
H	-1.948559	-0.138545	-2.913781
C	-3.849102	0.635591	-3.593743
H	-3.587629	0.559034	-4.647824
C	-5.097873	1.137082	-3.212298
H	-5.816983	1.450463	-3.967281
C	-5.414395	1.245953	-1.851556
H	-6.380485	1.643602	-1.545228
C	-4.490262	0.850195	-0.880529
H	-4.745328	0.964827	0.171425
C	-2.717400	-1.674542	0.837553
C	-1.859897	-2.475954	1.610675
H	-0.798503	-2.233097	1.655523
C	-2.349189	-3.586975	2.304544
H	-1.669358	-4.192529	2.901784
C	-3.705301	-3.925468	2.219640
H	-4.088151	-4.795734	2.750153
C	-4.563446	-3.141861	1.439581
H	-5.617636	-3.402169	1.358310
C	-4.074399	-2.020851	0.757854
H	-4.759656	-1.434417	0.150824
H	-0.571529	-1.891088	-3.028451
C	0.597769	-2.810184	-1.511527
C	-1.901376	-3.245077	-1.948791
C	0.767369	-3.879499	-0.559313
C	1.751012	-2.003081	-1.819152
C	2.999867	-2.288354	-1.219206
C	1.987064	-4.140394	0.011070
C	3.127073	-3.336113	-0.313861
H	1.739716	-1.357242	-2.699429
H	3.872138	-1.699150	-1.492179
H	-0.083202	-4.509998	-0.312273
H	2.099733	-4.965774	0.711377
C	4.394154	-3.624596	0.279297
H	-2.039227	-3.672302	-0.951567
H	-2.797463	-2.658447	-2.179553
H	-1.861222	-4.076595	-2.669242
N	5.428712	-3.868250	0.771197

8(T_1) E_{tot} = -1914.7472 a.u.

Rh	0.148332	-0.847044	-0.482798
P	1.279681	1.254417	-0.030250
P	-2.164222	-0.447045	-0.050440
C	0.032532	-2.914988	-1.148355
C	0.870358	2.064078	1.581863
C	0.939833	1.255568	2.731381
H	1.235956	0.209441	2.642979
C	0.646936	1.779155	3.993640
H	0.715272	1.139685	4.872069
C	0.261223	3.119754	4.124668
H	0.026368	3.529076	5.105715
C	0.174128	3.926573	2.985202
H	-0.131969	4.967615	3.075450
C	0.479333	3.404056	1.721758
H	0.401197	4.048586	0.849119
C	1.031276	2.557010	-1.323482
C	0.120607	2.309965	-2.360687
H	-0.442840	1.377124	-2.368359
C	-0.057071	3.240247	-3.392429
H	-0.764561	3.031507	-4.193160
C	0.677446	4.430494	-3.394302
H	0.542425	5.155364	-4.195356
C	1.598131	4.681877	-2.367172
H	2.180368	5.601830	-2.368839
C	1.778501	3.748752	-1.342804
H	2.515627	3.944297	-0.565311
C	3.140730	1.229418	0.010068
C	3.909151	1.614439	1.119526
H	3.425619	1.962142	2.029747
C	5.308709	1.569082	1.064862
H	5.889632	1.873184	1.934304
C	5.957727	1.147048	-0.100054
H	7.045149	1.117902	-0.143104
C	5.196506	0.759546	-1.211321
H	5.689590	0.425669	-2.122849
C	3.801450	0.790364	-1.151596
H	3.221985	0.473738	-2.018680
C	-2.843103	1.187480	0.520240
C	-3.235405	1.434069	1.845418
H	-3.182150	0.643101	2.590238
C	-3.718614	2.693655	2.223330
H	-4.024650	2.862826	3.254607
C	-3.815607	3.725560	1.284208
H	-4.195225	4.702682	1.578194
C	-3.423314	3.488842	-0.040372
H	-3.496912	4.281550	-0.783301
C	-2.938484	2.233908	-0.416348
H	-2.651367	2.067847	-1.453257
C	-3.337630	-0.807023	-1.437343
C	-2.831830	-1.365438	-2.619897
H	-1.768412	-1.583202	-2.692695
C	-3.677803	-1.633144	-3.703142
H	-3.267787	-2.062143	-4.615864
C	-5.043344	-1.344888	-3.612254
H	-5.703857	-1.551148	-4.452833
C	-5.557498	-0.780204	-2.436724
H	-6.618421	-0.547852	-2.361148
C	-4.710489	-0.507754	-1.358973
H	-5.121031	-0.050621	-0.460196
C	-2.667940	-1.572919	1.329859
C	-1.803015	-1.650413	2.436235
H	-0.893660	-1.050416	2.456504
C	-2.094151	-2.490802	3.515086
H	-1.414857	-2.533284	4.364854
C	-3.250807	-3.279892	3.496289
H	-3.476804	-3.941322	4.331012
C	-4.110739	-3.217584	2.393588
H	-5.008927	-3.832364	2.366898
C	-3.823675	-2.367745	1.318484
H	-4.501100	-2.342664	0.467908
H	-0.080693	-2.834140	-2.236908
C	1.425302	-2.989138	-0.765768
C	-0.963511	-3.883545	-0.536431
C	1.853438	-3.455110	0.528058
C	2.472576	-2.664056	-1.694086
C	3.809129	-2.769137	-1.365333
C	3.189682	-3.551242	0.864467
C	4.201395	-3.205756	-0.070027
H	2.194915	-2.343617	-2.698388
H	4.571694	-2.522175	-2.101656
H	1.105351	-3.741439	1.264631
H	3.475324	-3.904241	1.854465
C	5.573504	-3.313642	0.276284
H	-0.989710	-3.832797	0.557895
H	-1.978477	-3.664163	-0.886941
H	-0.735934	-4.926333	-0.813948
N	6.710193	-3.402307	0.562255
